# The Zr_8_O_6_ Secondary Building Unit and Porphyrin Linker Catalyze Light‐Driven H_2_ Evolution in Porphyrin‐Based Metal Organic Frameworks

**DOI:** 10.1002/cssc.202500372

**Published:** 2025-05-12

**Authors:** Subrata Mandal, Robert Leiter, Johannes Biskupek, Ute Kaiser, Andrea Pannwitz

**Affiliations:** ^1^ Institut für Anorganische Chemie I Universität Ulm Albert‐Einstein‐Allee 11 89081 Ulm Germany; ^2^ Central Facility of Electron Microscopy Electron Microscopy Group of Material Science University of Ulm Albert‐Einstein‐Allee 11 89081 Ulm Germany; ^3^ Institut für Anorganische und Analytische Chemie Friedrich‐Schiller‐Universität Jena Humboldtstr. 8 07743 Jena Germany; ^4^ Center for Energy and Environmental Chemistry Jena (CEEC) Friedrich‐Schiller‐Universität Jena Philosophenweg 7a 07743 Jena Germany; ^5^ Helmholtz Institute for Polymers in Energy Applications Jena (HIPOLE Jena) Helmholtz‐Zentrum Berlin für Materialien und Energie (HZB) Lessingstraße 12–14 07743 Jena Germany

**Keywords:** H_2_ evolutions, MOF, photocatalysis, porous coordination networks, Zr_8_O_6_

## Abstract

The four Zr_8_O_6_‐based metal‐organic frameworks (MOFs), porous coordination network (PCN) 221 M, comprising earth‐abundant metalloporphyrin tetracarboxylate (M‐TCPP, M: 2 H, Zn, Ni, and a mixture of 1:1 Zn and Ni), are investigated for light‐driven H_2_ evolution reaction (HER) in water. Under the irradiation of a 405 nm light emitting diode source and in the presence of triethanolamine (TEOA) as a sacrificial electron donor, the photocatalytic HER activity of PCN 221 varies with the metal center in the porphyrin linker. Among the tested MOFs, the Zn‐porphyrin derivative (PCN 221 Zn) produces H_2_ at a TON = 4, which is about seven times greater than that of homogeneous Zn‐TCPP (0.6) and superior to its 2 H (2.7), Ni (0.40), and ZnNi (1.6) analogs. Detailed photochemical studies via time‐resolved and steady‐state spectroscopy reveal two distinct charge transfer pathways: Direct H_2_ evolution from Zn‐TCPP itself, and electron transfer from the Zn‐TCPP photosensitizer to the Zr_8_O_6_ SBU catalytic sites. The improved HER performance of PCN 221 Zn is attributed to its favorable features, such as optical absorption, excited‐state properties, and charge separation dynamics, as well as the coordination of TEOA. This study provides fundamental insights into the design of MOF‐based heterogeneous photocatalysts exploiting earth‐abundant metal‐based porphyrin for solar fuel generation.

## Graphical abstract

Zr_8_O_6_‐based metal–organic frameworks  (porous coordination network PCN 221 M, M = 2 H, Zn, Ni, ZnNi) with metalloporphyrin linkers are explored for H_2_ evolution under 405 nm  light emitting diode irradiation. PCN 221 Zn exhibits the highest activity with a turnover number (TON) of 4 via dual charge transfer pathways. Spectroscopy revealed Zn‐TCPP acts as a direct HER site and an electron donor to Zr_8_O_6_.

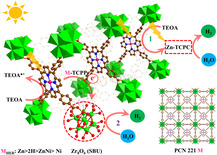

## 1 Introduction

The increase in global demand for sustainable energy requires the development of new energy conversion strategies. Photocatalytic H_2_ production through water splitting is one of the promising paths to transform and store solar energy as chemical fuels.^[^ ^1^ ^]^

A large variety of compounds have been used to split water into hydrogen including metal complex‐based homogenous photocatalysts,^[^ ^2^ ^]^ and noble metal nanoparticles,^[^ ^3^ ^]^ organic polymers,^[^ ^4^ ^]^ and inorganic semiconductors, as well as their composite materials in heterogeneous photocatalysts.^[^ ^5^, ^6^, ^7^ ^]^ However, developing efficient and robust photocatalysts to accelerate water splitting and achieve high hydrogen production rates is still a critical challenge. Recently, metal–organic frameworks (MOFs) have emerged as a new group of coordination frameworks and an ideal platform for solar energy conversion because of many advantages, such as ultrahigh surface areas, well‐developed pore networks, tunable chemical constituents, and semiconductor‐like properties.^[^ ^8^, ^9^, ^10^, ^11^, ^12^, ^13^, ^14^ ^]^ Notably, an MOF used for light‐driven H_2_ production should have the following features: 1) Strong light absorption. 2) Efficient charge transfer or separation. 3) Accessible catalytic sites. 4) High stability. While some MOFs fulfill these criteria,^[^ ^10^, ^11^, ^15^, ^16^ ^]^ their charge transfer dynamics and catalytic sites often remain elusive due to diverse coordination environments and multiple photocatalytic channels. To strategically improve the MOF photocatalyst design in the future, a deeper understanding of light‐driven charge transfer dynamics in MOFs is required.^[^ ^12^, ^17^, ^18^ ^]^

Porphyrins are promising photosensitizers for solar energy collection in photocatalytic energy conversion because of their excellent light‐harvesting capability, long excited‐state lifetime, and good stability.^[^ ^19^ ^]^ They are used in homogeneous photocatalysis for a wide range of applications, such as photocatalytic degradation of pollutants, hydrogen production, and carbon dioxide reduction.^[^ ^20^, ^21^ ^]^ Functionalization with carboxylates (—COOH) at their peripheral sites makes them an interesting molecular building block for porous MOF materials with various metal or metal‐oxo cluster‐based secondary building units (SBUs) (Zr, Al, Ce, Ti, Hf, etc.).^[^ ^22^, ^23^ ^]^ In addition, the porphyrin center can coordinate various functional metal ions, which can serve as active sites for catalysis.^[^ ^24^, ^25^ ^]^

For example, the SBU of Al(III)^[^ ^26^ ^]^ and Ti(IV)^[^ ^27^ ^]^ in porphyrin‐based MOFs can drive HER under visible light irradiation in the presence of colloidal platinum as the cocatalyst. Additionally, insertion of the noble metal single atoms, such as Pt(II),^[^ ^28^ ^]^ (Ir, Pt, Ru, Au, and Pd),^[^ ^29^ ^]^ Pt^[^ ^30^ ^]^ to the porphyrin center or colloidal NPs in association with the insertion of Pd (II), like a single atom into the porphyrin center^[^ ^31^ ^]^ by post‐synthetic modification, has improved the HER efficiency of such types of MOFs with Al, Zr, and Cu‐based SBUs. Alternatively, incorporating active metal complexes, such as biomimetic diiron complexes, at the linker as catalytic sites,^[^ ^32^ ^]^ HER activity has been improved. In another study, by developing Ru (III) SBU‐based porphyrin MOF, efficient visible‐light‐driven HER is achieved in neutral water.^[^ ^33^ ^]^

Taking into account these examples, the development of porphyrin‐based photocatalytic MOFs is focused on the introduction of an active precious metal‐based colloidal cocatalyst or single atom inserted into a porphyrin cavity or by developing novel MOFs of the precious metal‐oxo cluster, which serve as catalytic sites. However, a comprehensive understanding of the charge separation process and the specific active sites responsible for catalysis, which often differ among different MOF systems, is still insufficient, hindering the precise prediction of the mechanistic pathway. For example, single‐atom sites are claimed to be responsible for catalyzing the water reduction,^[^ ^22^ ^]^ whereas in another case, the SBU is responsible for water reduction.^[^ ^33^ ^]^

**Scheme 1:**
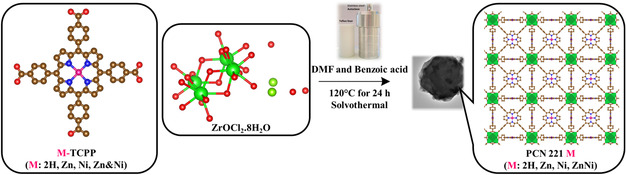
Schematic illustration of the synthesis of PCN 221M MOFs. The MOFs were prepared with slight modifications as reported earlier.^[^ ^37^ ^]^ (linker: M‐TCPP, SBU: Zr_8_O_6_, the MOF forms are abbreviated as PCN 221 M, where M: 2 H, Zn, and Ni, and ZnNi.

In this work, four Zr_8_O_6_‐based MOFs, so‐called porous coordination network, porous coordination network (PCN) 221 M, comprising of metalloporphyrin tetracarboxylate M‐TCPP linkers with M: 2 H, Zn (II), Ni (II), and a 1:1 mixture of Zn (II) and Ni(II) were prepared via a one‐step solvothermal synthesis using benzoic acid (BA) as a modulator in DMF solvent (**Scheme** **1**). Their light‐driven HER activities have been compared. It was expected that the mixed Ni‐Zn TCPP MOF (PCN 221 ZnNi) would yield the best HER result because Ni‐based porphyrins are the best molecular catalysts among the tested porphyrin linkers in homogeneous HER catalysis studies. Systematic spectroscopic investigations using time‐resolved and steady‐state spectroscopy were carried out to elucidate the electron transfer mechanism and the role of the Zr_8_O_6_‐based SBU within the MOFs.

## 2 Results and Discussion

### 2.1 Structural Characterizations

**Figure 1:**
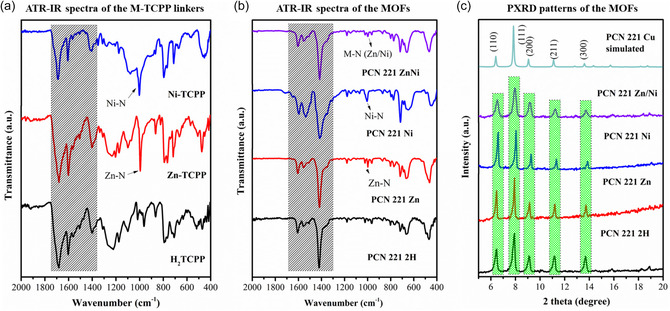
a,b) ATR‐IR spectra of the a) M*‐*TCPP linkers and b) the MOFs. c) PXRD patterns of the MOFs. The Simulated XRD pattern of PCN‐221 Cu was obtained from the crystallographic data reported earlier.^[^ ^37^ ^]^

The M‐TCPP linkers were synthesized according to the literature procedures as shown in Scheme S1, Supporting Information, and characterized by various techniques such as ^1^H nuclear magnetic resonance (NMR), attenuated total reflectance infrared (ATR‐IR), and UV–visible spectroscopy (Figure S1–S7, Supporting Information). Solvothermal reactions of Zr(IV)OCl_2_·8H_2_O with M‐TCPP linkers (M = 2 H, Zn, Ni, and 1:1 Zn and Ni), and BA in N, N‐dimethylformamide (DMF) at 120 °C yielded brown‐colored powders. Initially, the ATR‐IR spectra of the M‐TCPP linkers (**Figure** **1**a) and MOF samples (Figure 1b) were recorded. It can be seen from Figure 1a,b that the asymmetric vibrational absorption bands of C=O (1680–1690 cm^−1^) and C–OH (1262–1274 cm^−1^) of the M‐TCPP linkers are reduced and blue‐shifted compared to those in PCN 221 Ms. Additionally, a strong peak at around 1416–1420 cm^−1^ (—COO symmetric stretching band) appears for the MOFs PCN 221 2 H, PCN 221 Zn, PCN 221 Ni, and PCN 221 ZnNi, as shown in Figure 1b. This reflects that the carboxyl group of M‐TCPP coordinates to the Zr centers.^[^ ^34^ ^]^ Moreover, similar to the free Zn‐TCPP and Ni‐TCPP linkers (Figure 1a,b and S6a, Supporting Information), a symmetric metal–N stretching band at around 994–1002 cm^−1^ (994 cm^−1^ for Zn–N and 1002 cm^−1^ for Ni–N) is also observed for PCN 221 Zn, PCN 221 Ni, and PCN 221 ZnNi. This further reflects that the coordination environment of the N sites in the porphyrin moiety of PCN 221 Zn, PCN 221 Ni, and PCN 221 ZnNi is retained.^[^ ^35^, ^36^ ^]^ The powder XRD (PXRD) patterns of these MOF solids (Figure 1c) reveal that all samples are crystalline and appear to be single‐phase. The respective phases were identified by indexing their patterns with the reported topologies of PCN 221, as shown in Figure S8a, Supporting Information.^[^ ^37^ ^]^ Each phase (as shown in Figure 1c and Figure S8a, Supporting Information) exhibited characteristic reflection planes (110), (111), (200), (211), and (300) at angles 6.4°, 7.9°, 9.1°, 11.1°, and 13.7°, respectively, confirming that they are isostructural to the reported cubic phase of PCN 221(Co/Hf) and PCN 221(Cu/Zr) with the space group $Pm\bar{3}m$.^[^ ^37^ ^]^ Instead of Cu or Co in the porphyrin linker and Hf_8_O_6_ SBU, our metals, M, are 2 H, Zn, Ni, and a mixture of Zn and Ni, with Zr_8_O_6_ serving as the SBU. This unique topology of PCN 221 (Figure S8b, Supporting Information) has a periodic structure with a unit cell formula of Zr_8_O_6_(M‐TCPP)_3,_ where zirconium in the local environment of the MOF remains in a distorted octahedral coordination environment and is connected by three oxygen atoms from three carboxylates and three μ^4^‐oxygen atoms. Eight zirconium atoms connect six μ^4^‐oxygen atoms to form a Zr_8_O_6_ core, leading to an idealized Zr_8_ cube, in which the cubic vertices are occupied by zirconium atoms and the six faces are capped by six μ^4^‐oxygen atoms (Figure S8b, Supporting Information). Each edge of the Zr_8_ cube is bridged by a carboxylate from an M‐TCPP linker to give a [Zr_8_O_6_(CO_2_)_12_]^8+^ unit with O_h_ symmetry.

**Figure 2:**
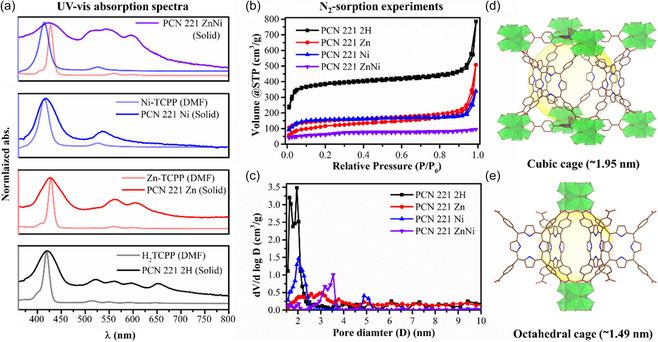
a) UV–Vis absorption spectra obtained from diffuse reflectance spectroscopy of the solid MOF; the background spectra represent the UV–Vis spectra acquired in the DMF solution of the respective M‐TCPP linker. b) N_2_ adsorption–desorption isotherms of the MOFs. c) Pore size distribution curves. Representation of two types of cavities, d) cubic (top) and e) octahedral (bottom), interconnected to form open channels in three dimensions of PCN 221 MOF. The structure depicted here was obtained from CCDC 925 058^[^ ^37^ ^]^ and was analyzed, and visualized using Dimond and Vesta software, respectively for enhanced clarity and precision.

Furthermore, solid‐state diffuse reflectance spectroscopy of the MOFs was carried out, and the acquired UV–vis absorption spectra are presented in **Figure** **2**a. For comparison, the UV–vis absorption spectra of their respective linkers in the DMF solution are also shown. The results demonstrate that all the MOFs retained the characteristic spectral features of their respective linkers (see Supporting Information for a detailed discussion on the linkers). For instance, the Soret bands and Q‐bands of the M‐TCPP linkers appeared at their expected wavelengths, confirming that no metal leaching from the porphyrin occurred during synthesis. This conclusion is supported by the merging of the four Q‐bands (522, 561, 599, and 652 nm) of the PCN 221 2 H spectrum into two Q‐bands (563 nm and 606 nm) in the PCN 221 Zn spectrum and further into a single Q‐band (537 nm) in the PCN 221 Ni spectrum. Notably, in solution, the Zn‐TCPP linker exhibits the highest molar absorption coefficient (ε: 311 785 L mol^−1^ cm^−1^), followed by H_2_TCPP (285 513 L mol^−1^ cm^−1^) and Ni‐TCPP (41 910 L mol^−1^ cm^−1^) (Figure S7, Supporting Information). The observed variations in absorption properties, along with the trend in the number of Q‐bands across both the homogeneous M‐TCPP linkers and the MOFs, arise from changes in porphyrin symmetry and planarity induced by the metal‐N coordination environment. Specifically, H_2_TCPP, initially exhibiting D_2_h symmetry, transforms into a more symmetric and planar D_4_h configuration upon coordination with Zn(II), maintaining its structural integrity.^[^ ^38^, ^39^, ^40^ ^]^ In contrast, Ni(II) favors a nonplanar S_4_ conformer, characterized by an out‐of‐plane geometry.^[^ ^41^, ^42^ ^]^ This structural distortion in Ni‐TCPP leads to a blue shift and a reduction in molar absorption.^[^ ^38^, ^40^, ^41^ ^]^ In the mixed metal‐MOF, PCN 221 ZnNi, the presence of both Ni‐TCPP and Zn‐TCPP linkers is evidenced by three Q‐bands, which are the addition of the respective Q‐bands in Ni‐TCPP and Zn‐TCPP, as can be seen in Figure 2a. Upon incorporation of the porphyrin linkers into the respective MOF structure, a broadening and slight redshift of the absorption bands were observed, probably due to the periodic arrangement of the linkers in the solid‐state framework and the coordination of the —COO^−^ groups to the Zr centers, which typically causes a bathochromic shift.^[^ ^43^ ^]^ A hypsochromic shift of the absorption bands in the MOFs would have been expected due to the dihedral angle (90°) between the porphyrin plane and the peripheral benzene dicarboxylate group.^[^ ^44^ ^]^ However, this effect seems to be minor in the investigated PCN 221 Ms. Importantly, no new absorption bands were observed in these MOFs, indicating that the absorption properties are predominantly determined by the molecular properties of the linker unit. Among the PCN 221 M and PCN 221 Zn is the most suitable light harvester. The typical optical energy gap (*E*_g_ = E_HOMO‐LUMO_) for the MOFs was estimated from their lower energy absorption edges, using the Kubelka–Munk method,^[^ ^45^, ^46^ ^]^ The calculated *E*_g_ are 1.79 eV for PCN 221 2 H, 1.85 eV for PCN 221 Zn, 1.90 eV for PCN 221 ZnNi, and 2.10 eV for PCN 221 Ni (Figure S9, Supporting Information). Metalation, progressing from no metal to Ni via Zn, increases the optical energy gap.

The porosity and surface area of the four PCN 221 MOFs were quantified by N_2_ sorption experiments at 77 K. In Figure 2b, all MOFs exhibited a type I isotherm at the relative pressure (P/P_0_) < 0.1, indicating permanent micropores, and a type IV isotherm with an H3 hysteresis loop at P/P_0_ > 0.9, suggesting a mesoporous nature due to agglomerated MOF nanoparticles or linker defects.^[^ ^47^, ^48^ ^]^ The physical parameters, including the Brunauer–Emmett–Teller (BET) surface area, total pore volume, and average pore diameters, were calculated and are shown in Table S1, Supporting Information. The BET surface areas are 1494, 348, 578, and 236 m^2^ g^−1^ for PCN 221 2 H, PCN 221 Zn, PCN 221 Ni, and PCN 221 ZnNi, respectively, and the total pore volume obtained from the DFT (Density Functional Theory) model of PCN 221 2 H is 1.044 cm^3^ g^−1^ and highest among the PCN 221 M materials with 0.67, 0.46 cm^3^ g^−1^, 0.14 cm^3^ g^−1^ for M = Zn, Ni, and ZnNi, respectively. The decrease in surface area and pore volume upon incorporation of M = Zn, Ni, and ZnNi is very typical for the presence of metal sites in the porphyrin's center, as metal–N interactions among Zn‐, Ni‐, and Zn/Ni‐based MOFs can lead to structural distortions, altering pore size distribution and adsorption properties.^[^ ^37^, ^49^, ^50^ ^]^ Considering the pore width calculated from the DFT model, PCN 221 2 H exhibited micropores at around 1.6 and 1.9 nm, as evident from the pore size distribution curve in Figure 2c. The pore width matches very closely the octahedral and cubic pores in the PCN 221 crystal (Figure 2d,e).^[^ ^37^ ^]^ The decrease in micropore volume and the increase in average pore diameters (2–4 nm, mesoporous regime) in PCN 221 Zn, PCN 221 Ni, and PCN 221 ZnNi compared to PCN 221 2 H may not solely result from metalation but could also come from several factors.^[^ ^50^, ^51^, ^52^ ^]^ The reduced particle size and thinner 2D‐like structure of the PCN 221 M (Zn, Ni, and ZnNi) lead to a significant loss of intrinsic microporosity, decreasing the N_2_‐BET surface area and pore volume. Additionally, the Zn and Ni within the porphyrin pocket may partially block the pores, limiting available free volume for gas adsorption. The increased density of the metalated MOFs further contributes to a lower specific surface area, while linker defects and interactions with additives (e.g., BA, formic acid (HCOOH)) and solvent (DMF) can induce mesoporosity and micropore blocking. Despite these modifications, the remaining micropores and mesopores in these PCN‐221 MOFs ensure sufficient exposure of active sites for catalysis.

**Figure 3:**
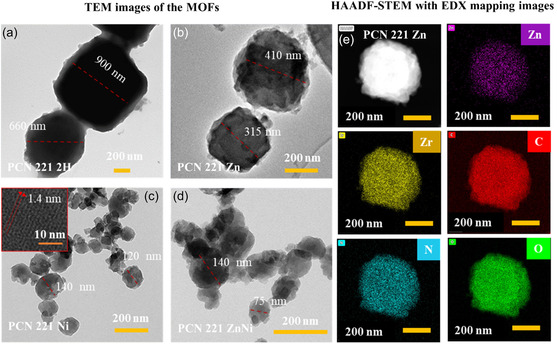
a–d) TEM images of MOFs: a) PCN 221 2 H, b) PCN 221 Zn, c) PCN 221 Ni (inset: magnified HRTEM image showing lattice fringes), and d) PCN 221 ZnNi. e) High‐angle annular dark field scanning transmission electron microscopic (HAADF‐STEM) image corresponding with EDX element mapping images of PCN 221 Zn. The purple, yellow, red, cyan, and green signals represent Zn, Zr, C, N, and O, respectively.

Furthermore, transmission electron microscopy (TEM) of the PCN 221 MOFs was carried out, and the respective images in **Figure** **3**a–d clearly show that all the MOFs are crystallized into pseudospherical morphology, which is a characteristic of the PCN 221 structure and in line with the morphology of some reported PCN 221 analogs.^[^ ^37^, ^53^ ^]^ The average size and crystallinity varied among PCN 221 MOFs. The PCN 221 2 H displayed particles with an average diameter of 500 nm, while PCN 221 Zn, PCN 221 Ni, and PCN 221 ZnNi showed average diameters of 400, 200, and 100 nm, respectively, indicating smaller sizes and relatively lower crystallinity. These differences likely originate from the reaction kinetics between Zr(IV) and M‐TCPP linkers, as well as crystal growth during synthesis. Factors such as the acidity, solubility, and geometry of the M‐TCPP linkers, which change upon metalation, can significantly influence these properties.^[^ ^54^ ^]^ High‐angle annular dark‐field scanning transmission electron microscopy (HAADF‐STEM) images together with corresponding energy dispersive X‐ray (EDX) element mappings of PCN 221 Zn are shown in Figure 3e and S10–S13, Supporting Information, for PCN 221 M (M = 2 H, Ni, and ZnNi), respectively. The HAADF‐STEM and EDX images clearly show the elements Zr, C, N, and O, which are distributed over the entire structure of each MOF. The signals for Zn were detected in PCN 221 Zn and PCN 221 ZnNi, and Ni was detected in PCN 221 Ni and PCN 221 ZnNi. Unlike C, N, O, and Zr in the PCN 221 M (Zn, Ni, and ZnNi), the Zn and/or Ni metals appear to be more spatially isolated. This could be attributed to the separation of individual metal centers within each porphyrin unit, enforced by the framework structure, or the low relative composition of these metals in the material. The EDX results of all MOFs (Figure 3 and S10–S13 and Table S2, Supporting Information) confirmed the presence of respective expected elements in all MOFs. The experimentally determined Zr/N ratios for the PCN 221 2 H and PCN 221 Zn are 0.69 and 0.57, respectively. These ratios are very close to the expected ideal ratio Zr/N = 0.67 according to the formula [Zr_8_O_6_][(M‐TCPP)_3_], M = Zn or 2 H, for PCN 221. For the other two MOFs, PCN 221 Ni and PCN 221 ZnNi, high Zr/N ratios of 1.22 and 1.53 were obtained, indicating the Ni‐containing MOFs deviate from the ideal formula, either due to additional solvent from the synthesis or due to linker vacancies. The experimentally determined Zr/M ratios for the PCN 221 Ms (Zn, Ni, and ZnNi) were between 2.45 and 2.71, which matches well with the ideal ratio of Zr/M = 2.67. These results also indicated that almost all sites of the porphyrins are occupied by M, and no significant leaching has occurred during the synthesis. To quantify the content of potential additional solvent in the crystal structure, we performed ^1^ H NMR analysis on digested MOFs,^[^ ^55^, ^56^ ^]^ similar to a reported protocol. As also reported from these studies,^[^ ^55^, ^56^ ^]^ the nanostructured MOFs contained the additive BA, HCOOH (hydrolyzed product of DMF), and the DMF solvent from synthesis as guest molecules (Figure S14, Supporting Information). The actual molar ratios of M*‐*TCPP, BA, HCOOH, and DMF were calculated to obtain the real formulas [Zr_8_O_6_ (M‐TCPP)_*x*_(BA)_*y*_(HCOO)_*z*_]·*n* DMF, where *x*, *y*, z, and *n* are the molar equivalents. The respective formulas are summarized in **Table** **1** (see Supporting Information page S13 and Table S3, Supporting Information, for experimental details and calculation).

**Table 1:** Calculated formulas of the MOFs, according to ^1^ H NMR.

Sample	Calculated formula of the MOF [Zr_8_O_6_ (M‐TCPP)_*x*_(BA)_*y*_(HCOO)_*z*_]·*n* DMF
**PCN 221 2 H**	[Zr_8_O_6_ (H_2_TCPP)_1.71_ (BA)_3.76_ (HCOO)_1.40_]·0.27 DMF
**PCN 221 Zn**	[Zr_8_O_6_ (Zn‐TCPP)_1.51_ (BA)_4.73_ (HCOO)_1.23_]·0.24 DMF
**PCN 221 Ni**	[Zr_8_O_6_ (Ni‐TCPP)_1.62_ (BA)_4.74_ (HCOO)_0.78_]·0.78 DMF
**PCN 221 ZnNi**	[Zr_8_O_6_ (Zn/Ni TCPP)_2.04_ (BA)_3.67_ (HCOO)_0.16_]·0.49 DMF or [Zr_8_O_6_ (Zn‐TCPP)_0.24_ (Ni‐TCPP)_1.80_ (BA)_3.67_(HCOO)_0.16_]·0.49 DMFa)

a) The ratio of Zn and Ni in PCN 221 ZnNi MOF was calculated from the EDX data summarized in Table S2, Supporting Information.

**Figure 4:**
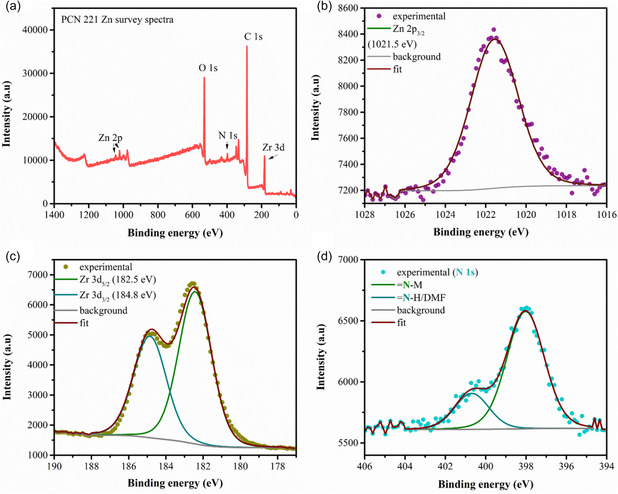
XPS of PCN 221 Zn MOF: a) survey, b) Zn 2p_3/2_, c) Zr 3d, and d) N1s.

The valence states of Zr, Zn, C, O, and N in the representative MOF PCN‐221 were analyzed by X‐ray photoelectron spectroscopy (XPS). The full survey spectrum is shown in **Figure** **4**a, showing all expected elements. Figure 4b shows the peak at 1021.5 eV, which corresponds to Zn 2p_3/2_ and is in line with a Zn(II) in the Zn‐N coordination environment.^[^ ^57^ ^]^ Figure 4c shows the peaks at 182.5 eV and 184.8 eV, which are assigned to Zr 3d_5/2_ and Zr 3d_3/2_ and confirm the existence of the Zr (IV) node in the PCN 221 Zn.^[^ ^37^ ^]^ Intense signals for C 1s at 284.8 eV (—C=C/—C=N) and 288.72 eV (—COO), along with a broad O 1s signal around 531.7 eV (—COO and Zr‐O), are also observed (Figure S15, Supporting Information). The N 1s region (395.5 − 402.1 eV, Figure 4d) can be deconvoluted into two peaks for N‐Zn (≈397.9 eV) within the Zn‐TCPP linker and the uncoordinated N—H or N—C from DMF (≈400.6 eV), in line with previous studies.^[^ ^22^, ^58^ ^]^

### 2.2 Photocatalytic HER

**Figure 5:**
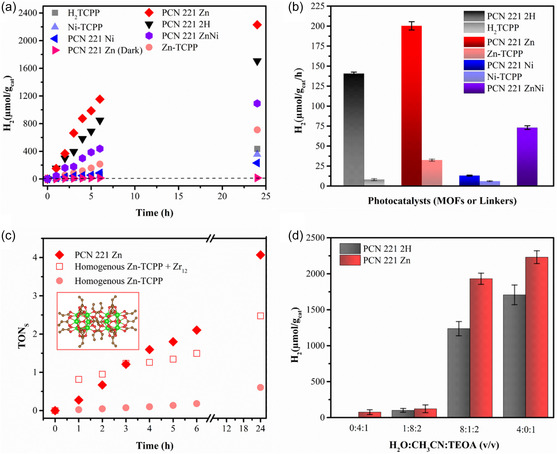
a) H_2_ production (μmol/g_cat_ vs. time) for 24 h, and b) comparison of H_2_ production rates (μmol/g_cat_/h) of the PCN 221 M MOFs with their corresponding homogeneous M‐TCPP linkers within 6 h. Reaction conditions: ≈1 mg of MOF/or ≈0.75–0.90 mg of M‐TCPP linkers as a photocatalyst; solvent: deaerated H_2_O: TEOA (4:1 v/v); *λ*: 405 nm LED source. c) Comparison of H_2_ production (TON_S_ vs. time) up to 24 h for (1) Zn‐TCPP alone [1.04 μmol], (2) Zn‐TCPP [0.44 μmol] + Zr_12_ [3.40 μmol], and (3) PCN 221 Zn MOF under the following reaction conditions: deaerated H_2_O: TEOA (4:1 v/v); *λ*: 405 nm LED source. d) H_2_ production (μmol/g_cat_ vs. time) of the PCN 221 Zn and PCN 221 2 H MOFs under different ratios of solvent and donor mixture (H_2_O: CH_3_CN: TEOA v/v) after 24 h of light irradiation.

Light‐driven HER by the MOFs was carried out with TEOA as a sacrificial donor in deaerated water and is depicted in **Figure** **5**. Irradiation occurred for 24 h by light emitting diode (LED) light source at 405 nm, matching the Soret band of the porphyrins. The amount of generated H_2_ gas was measured in the headspace of the reaction vials using gas chromatography (GC) and manual injections at each time interval (see experimental part for more details). The photocatalysis data, shown in Figure 5a, can be fitted linearly in the first 6 h (see Figure S16a, Supporting Information), yielding the respective initial reaction rates reported *vide infra*. Over the course of 24 h, the reaction rate decreases. The control experiments for the PCN 221 Zn without light irradiation yielded no detectable H_2_ amounts, referring light source to be responsible for activating the MOFs for driving the HER. The PCN 221 Zn produced the highest amount of H_2_ with (2230 ± 89 μmol g^−1^ during 24 h at an initial reaction rate of 200 ± 5.2 μmol g^−1 ^h^−1^), followed by PCN 221 2 H with 1706 ± 136 μmol g^−1^ (140 ± 1.8 μmol g^−1 ^h^−1^). The Ni‐containing MOFs yielded the poorest performance: PCN 221 Ni with 230 μmol g^−1^ (initial rate of 13 ± 0.6 μmol g^−1^ h^−1^) and PCN 221 ZnNi with 1091 μmol g^−1^ (initial rate of 73 ± 2.3 μmol g^−1^ h^−1^). These results are surprising because, under homogeneous conditions, the Ni‐based porphyrin is known as an efficient molecular catalyst for HER.^[^ ^59^ ^]^ Additionally, Zn‐porphyrins and metal‐free porphyrins are known for their role as photosensitizers.^[^ ^33^ ^]^ It was therefore initially expected that the mixed‐linker MOF PCN 221 ZnNi would yield the highest activity for light‐driven HER. However, in the PCN 221 MOFs, the presence of Ni in the porphyrin linkers seems to significantly reduce the activity of light‐driven HER, and while no photocatalytic activity was expected from the photosensitizing Zn‐porphyrin linker, the pure Zn‐porphyrin MOF yielded the highest activity (almost 1.3, and 10‐fold higher than PCN 221 2 H and PCN 221 Ni, respectively) and the mixed linker MOF, PCN 221 ZnNi, yielded just half the amount of H_2_ compared to the uniform PCN 221 Zn.

To test the role of the porphyrin linkers in light‐driven HER, the pure linkers were subjected to the photocatalysis conditions. Figure 5a shows that small amounts of H_2_ were produced by the linkers under homogeneous conditions. The H_2_ production rates for H_2_TCPP, Zn‐TCPP, and Ni‐TCPP were 8 ± 1.2, 32 ± 1.1, and 6 ± 0.6 μmol g^−1 ^h^−1^, respectively, and are ≈17, 6, and 25 times slower compared with the respective MOFs (see Figure 5b). To better compare the molecular activity, the turnover numbers (TONs) were compared.^[^ ^60^ ^]^ The TON is independent of the molecular weight and is calculated via TON = n_H2_/n_cat_, where n_H2_ is the amount of product in mol and n_cat_ is the amount of photocatalyst in mol. By assuming the porphyrin (S: TCPP unit) is the photocatalyst in the MOF and by using the molecular formulas from Table 1, the following TON_S_ were calculated. The active MOFs achieved significantly higher TON_S_ with TON_PCN 221 Zn_ =4.0, TON_PCN 221 2 H_ = 2.7, and TON_PCN 221 ZnNi_ = 1.6 (data plots see Figure S16b, Supporting Information). On the other hand, the TONs calculated for the homogenous linkers were smaller than unity in all cases, with the highest performer being Zn‐TCPP with TON_Zn‐TCPP_ = 0.6, maintaining the trend Zn > 2 H (or H_2_) > Ni and ZnNi. These results indicate that the molecular porphyrin alone cannot explain the increased activity of the MOFs and that either the 3D arrangement or the SBU of the MOF might play an important role in the photocatalysis.

To elucidate the role of the 3D arrangement within the MOF structure and test the catalytic activity of the SBU Zr‐cluster, the SBUs were replaced by the water‐soluble Zr_12_ cluster [Zr_6_(OH)_4_O_4_(OAcr)_12_]_2_·6 AcrOH (OAcr = acrylate), which has a similar structure as the SBU cluster Zr_8_O_6_ (see Figure 5c, synthesis and characterization in the Supporting Information on page S16 and 17 and Figure S17, Supporting Information).^[^ ^61^, ^62^ ^]^ Light‐driven HER was monitored over time up to 24 h and is shown in Figure S18a, Supporting Information, and yielded 600 μmol g^−1^ H_2_ for 0.44 μmol of Zn‐TCPP alone and 2800 μmol g^−1^ for Zn‐TCPP in the presence of ≈8 eqv. of Zr_12_ (referenced to the weight of Zn‐TCPP), and 2230 μmol g^−1^ for PCN 221 Zn. These performances correspond to the following TONs: TON_Zn‐TCPP_ = 0.6, TON_Zn‐TCPP+Zr12_ = 2.5 (referenced to Zn‐TCPP), and TON_PCN 221 Zn_ =4.0 (referenced to Zn‐TCPP) (Figure 5c). To estimate the Zn‐TCPP content in PCN 221 Zn MOF, the actual formula [Zr_8_O_6_ (Zn‐TCPP)_1.69_ (BA)_5.24_] 1.35 DMF obtained from the ^1^H NMR analysis of the digested MOF is used. The significant enhancement of the photocatalytic activity of the Zn‐porphyrin in the presence of the water‐soluble Zr_12_ cluster supports the hypothesis that the Zr‐cluster acts as a catalyst in the MOF. The significant enhancements of light‐driven HER activity of the MOF versus the homogeneous mixture of Zn‐TCPP in the presence of Zr_12_ confirmed the important role of the hierarchical organization of photosensitizing linkers and the catalytically active SBUs, which can facilitate multielectron transfers to drive photocatalytic HER.^[^ ^63^ ^]^

To characterize the proton source, the water in the reaction mixture was replaced by the nonprotic solvent acetonitrile (CH_3_CN), resulting in only trace amounts of H_2_. In solvent mixtures H_2_O: CH_3_CN, the HER activity increased with increasing water content and was best at H_2_O: CH_3_CN = 1:0 (see Figure 5d). These results confirm that H_2_O is the source of H^+^. The role of TEOA was tested by reducing the amount of TEOA in the reaction mixture, which resulted in decreased H_2_ formation (see Figure S18b, Supporting Information). These data confirm that TEOA is limiting in the overall light‐driven HER and acts as an electron donor.

The Zn‐TCPP absorbs both via the intense, high‐energy Soret band at around 427 nm and via the less intense, lower‐energy Q bands at around 510‐600 nm. However, all data discussed above are measured only at the intense Soret band with a 405 nm light source (900 mW, with a current of 0.4 A and a voltage of 14 V). When changing the irradiation source to 530 nm (281 mW, with a current of 0.5 A and a voltage of 3.4 V), matching the Q bands, light‐driven HER is maintained with a reduced TON for the MOFs and the homogeneous M‐TCPP + Zr_12_ mixture (M = Zn, H_2_) (see Figure S19, Supporting Information), indicating that the lower energy Q bands of the porphyrins are indeed also active in light‐driven HER.

While the overall light‐driven HER activity is quite moderate with TON = 4.0 at the rate of 200 ± 5.2 μmol g^−1 ^h^−1^ for PCN 221 Zn, it is one order of magnitude better than some Al node‐based porphyrin MOFs with noble metal co‐catalysts (Al‐PMOFs/colloidal Pt and Al‐TCPP‐PtNPs).^[^ ^26^, ^28^ ^]^ While other MOFs, with precious metal‐based linkers and additional noble metal‐based cocatalysts (Pt@Pd‐PCN‐222(Hf), PCN 222 HNTM/Pt NPs, and Ultrathin PMOF/Pt, etc.),^[^ ^27^, ^29^, ^31^ ^]^ are although several orders of magnitude more efficient in light‐driven HER, but the here‐reported PCN 221 Zn is the only reported porphyrin‐based MOF based on earth‐abundant metals in light‐driven HER, as can be seen in **Table** **2**. The here‐reported PCN 221 Zn MOF can therefore be a cheap candidate for various photocatalytic applications. While the Zr_6_‐based PCN‐222 MOF has been reported to achieve an H_2_ evolution rate of 351.08 μmol g^−1^ h^−1^ with a Pt catalyst (Table 2), our attempt to synthesize PCN 222 Zn resulted in a mixed‐phase material with PCN 221 Zn. Despite its larger pores and expected catalytic benefits,^[^ ^64^, ^65^, ^66^ ^]^ this phase impurity prevents a conclusive comparison between Zr_8_‐ and Zr_6_‐based MOFs in this study.

**Table 2:** Summary of the photocatalytic HER with porphyrin‐based MOFs.

MOFs	M‐TCPP	SBU	Co‐catalyst	Sacrificial reagent	Power/hν	Efficiency [μmol g^−1^ h^−1^]	ref.
PCN 221 Zn PCN 221 2 H PCN 221 ZnNi PCN 221 Ni	Zn‐TCPP	Zr_8_O_6_	–	H_2_O:TEOA	LED source (900 mW) *λ* = 405 nm	200 ± 5.2 140 ± 1.8 73 ± 2.3 13 ± 0.6	This work
Al‐PMOF Al/Zn‐PMOF	H_2_TCPP Zn‐TCPP	[AlOH]_2_(DMF_3_(H_2_O)_2_)	Colloid. Pt	Aq. EDTA	300 W Xe UV–vis	200 100	[26]
Ultrathin PMOF	H_2_TCPP	Ti_17_O_6_	Pt	Aq. ascorbic acid	300 W Xe *λ* > 420 nm	8 520	[27]
Al‐TCPP‐PtNPs (NPs: nanoparticles) Al‐TCPP‐0.1Pt(II)	H_2_ TCPP Pt‐TCPP	Al(OH)O_4_	Pt NPs	CH_3_CN:H_2_O TEOA	300 W Xe *λ* > 380 nm	50 129	[28]
PCN 222 HNTM	IrTCPP Cl Pt‐TCPP^a^ Ir/Pt‐TCPP	Zr_6_(μ_3_‐O)_4_(μ_3_‐OH)_4_(OH)_4_ (H_2_O)_4_	–	CH_3_CN:H_2_O TEOA	300 W Xe *λ* > 400 nm	7 600 56 700 201900	[29]
2D SACs	Pt TCPP	Cu_2_(COO)_4_	Single atom Pt^0^	Aq. ascorbic acid	300 W Xe *λ* > 420 nm	11320	[30]
Pt@Pd‐PCN‐222(Hf)	Pd TCPP	Zr_6_(μ_3_‐O)_4_(μ_3_‐OH)_4_(OH)_4_(H_2_O)_4_	Pt	CH_3_CN:H_2_O:TEOA	300 W Xe *λ* ≥ 420 nm	22674	[31]
Ru‐TBP Ru‐TBP‐Zn	Ru TCPPCl ZnTCPP	Ru_2_O_4_ (DMF)(H_2_O)_2_	–	CH_3_CN:H_2_O:TEOA	230 W *λ*> 400 nm	130 240	[33]
PCN222‐H_2_/Pt0:1	H_2_TCPP Pt TCPP	Zr_6_(μ_3_‐O)_4_(μ_3_‐OH)_4_(OH)_4_(H_2_O)_4_	–	CH_3_CN:H_2_O:TEOA	Xe‐lamp *λ* ≥ 400 nm	351.08	[22]

**Figure 6:**
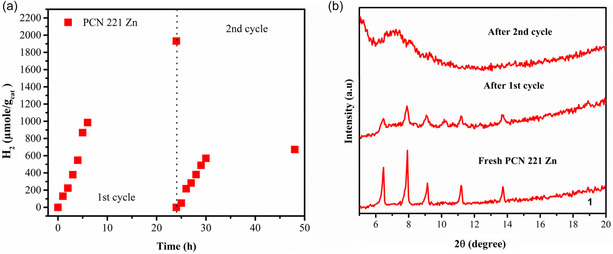
a) 1st and 2nd cycle of the PCN 221 Zn MOF for the photocatalytic production of H_2_ (μmol/g_cat_ vs. time) monitored for 48 h under the following reaction conditions: 1 mg of MOF, deaerated H_2_O: CH_3_CN: TEOA (8:1:2 v/v); *λ*: 405 nm LED source. For the 2nd cycle the solid was separated from the solution by centrifugation at 7000 rpm, washed with CH_3_CN (twice), and reirradiated (2nd cycle) with the deaerated H_2_O: CH_3_CN: TEOA (8:1:2 v/v). b) PXRD pattern of the PCN 221 Zn sample dried after each cycle, obtained from separate experiments.

The stability of MOFs was found to be limited to around 24 h. As this is a short period, the recyclability was tested. It was found that in an 8:1:2 v/v H_2_O/CH_3_CN/TEOA mixture, the MOF could be recovered and utilized for a 2nd cycle of light‐driven HER experiments (**Figure** **6**a). For that, the MOF was washed with ethanol after the 1st cycle and reused for the next cycle by adding an 8:1:2 v/v H_2_O/CH_3_CN/TEOA mixture. Notably, the MOF loses its activity by almost three times in the 2nd cycle, which can be attributed to the leaching of the linker into the solvent, which induces a loss of crystallinity as observed from the XRD measurements (Figure 6b). The basic pH might cause strong nucleophilic species such as OH^−^ and amines to compete with the coordination at the Zr–SBUs and thereby dissolve the MOF. Such structural collapsing is a common issue for MOF‐based photocatalysts and may take place chemically or during light irradiation.^[^ ^67^, ^68^, ^69^, ^70^ ^]^As a potential strategy for improving stability, such as optimizing the reaction conditions (a polar aprotic solvent/TEOA ratio with minimum water content), we conducted the HER experiments in a CH_3_CN: Water: TEOA (8:1:2 v/v) mixture instead of the CH_3_CN: Water: TEOA (1:8:2 v/v). Although the HER activity is quite low (120 μmol/g_cat_), it is almost 16 times lower. But under this condition, the MOF steadily performs HER for multiple cycles without losing the photocatalytic activity (see Figure S20a, Supporting Information) and crystallinity (see Figure S20b, Supporting Information). Modifying the cluster through hydrophobic functional groups can be an alternative strategy to gain the long‐term stability and retain the high activity of the photocatalyst under water/TEOA conditions.

### 2.3 Photophysical and Photochemical Processes

To elucidate the reasons for the surprising photocatalytic HER efficiency of PCN 221 Zn compared to the mixed‐metal MOF containing Ni‐based porphyrins, the electron transfer dynamics were resolved using steady‐state and time‐resolved emission spectroscopy and absorption, as summarized in **Table** **3** and as discussed vide infra.

**Table 3:** Table of photophysical characterization of the M‐TCPP linkers and PCN 221‐M.

Compounds	UV–vis absorption		Optical gap	Singlet state emission		Triplet state
	*λ* (ε)/nm [L mol^−1^ cm^−1^]		*E*_g _= E_HOMO‐LUMO_)/eV	[*λ*/nm]	[τ /ns]	[τ /μs] (%contribution)
	Soret band	Q band				
H_2_TCPP	419 (285513)	514 (14563), 549, 590, 645	–	650, 715	11.63	149 (8), 3356 (92)
Zn‐TCPP	427 (311785)	518, 559 (13397), 599	–	608, 659	2.07	167 (29), 911 (71)
Ni‐TCPP	415 (41910)	527 (3113)	–	550 (weak)	3.02	–
PCN 221 2 H	420a)	521, 561, 595, 653a)	1.79b)	654, 715	0.89	0.90 (26), 5.50 (74)c) 0.22 (19), 2.03 (81)d)
PCN 221 Zn	428a)	520, 563, 606a)	1.85b)	626, 658	0.52	‐, 12.50c) 0.37 (14), 5.54 (86)d)
PCN 221 Ni	418a)	537a)	2.10b)	–	–	–
PCN 221 ZnNi	424a)	518, 545, 595a)	1.88b)	660, 718	–	–

a) Absorption spectra acquired from the diffuse reflectance spectroscopy of the MOF in the solid state.

b) Optical energy gaps (*E*_g_) of the solids were estimated from the Kubelka Munk functions.

c) Measured triplet state lifetimes for the unmodified MOFs (PCN 221 2 H, and PCN 221 Zn) at *λ*_ex (pump) _= 410 nm.

d) Measured triplet state lifetimes for the PEG‐NH_2_ modified MOFs (PCN 221 2 H, and PCN 221 Zn) at *λ*_ex (pump) _= 425, and 450 nm, respectively.

Steady‐state and time‐resolved emission spectroscopy of the pure porphyrin ligands in organic solvents characterized the emission from the respective singlet excited states. It revealed a significant blue shift of the two emission features upon metalation from 650 and 715 nm for H_2_TCPP to 608 and 659 nm for Zn‐TCPP. Their lifetime were 11.63 and 2.07 ns, respectively (Figure S21, Supporting Information). Upon coordination with Ni, the porphyrin singlet state is almost completely quenched with a very weak emission maximum at 550 nm.

The respective triplet excited states can be probed by transient absorption spectroscopy, as shown in Figure S22, Supporting Information, and reported for porphyrins previously.^[^ ^24^ ^]^ It revealed two long‐lived species with positive absorption features at 445 and 460 nm for H_2_TCPP and Zn‐TCPP, respectively, in line with porphyrin triplet state spectra reported between 380–400 nm.^[^ ^71^ ^]^ Their lifetime was composed of two components, with lifetimes on the order of around 150 μs and a long‐lived component on the ms‐timescale. The Ni‐containing linker, Ni‐TCPP, however, did not show any long‐lived excited state on the timescale of the transient absorption experiment with a resolution of approx. 10 ns, which is in line with previous reports on Ni‐porphyrins.^[^ ^24^, ^72^, ^73^ ^]^ The lack of a long‐lived excited state on the Ni‐porphyrin linker supports that it is a poor photosensitizer.

**Figure 7:**
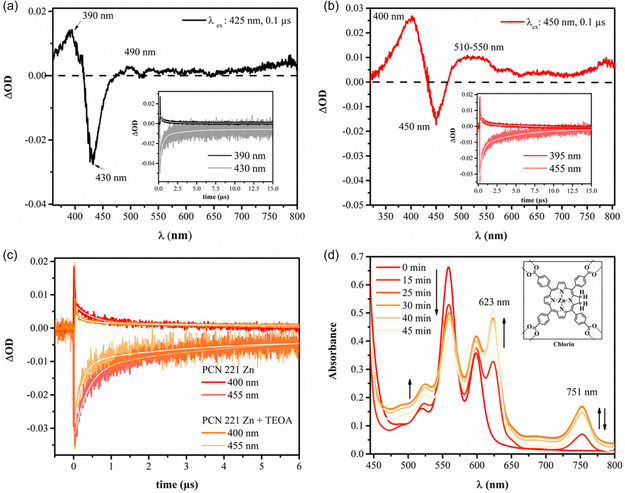
Transient absorption spectra of the PEG‐NH_2_ modified a) PCN 221 2 H and b) PCN 221 Zn MOF in deaerated DMF at a time delay of 100 ns, and each inset shows corresponding kinetic traces of excited state absorption (ESA) and ground state bleaching (GSB). c) A comparison of the kinetic traces for the ESA and GSB of PEG‐NH_2_ modified PCN 221 Zn in a deaerated DMF solution in the presence and absence of TEOA. d) Steady‐state UV–vis absorption spectra of the PCN 221 Zn under the actual photocatalytic conditions.

When integrating the porphyrin linkers into the MOF structures, the porphyrin absorption features are qualitatively maintained, as discussed above. Considering the excited states, the porphyrins’ singlet and triplet excited states were quenched completely for PCN 221 Ni but persisted in PCN 221 2 H and in PCN 221 Zn (Table 3). The respective singlet excited state emission showed very small band shifts except for the high‐intensity emission band of the Zn‐porphyrin, which was reduced in intensity and red‐shifted by approx. 20 nm in the solid MOF. However, this might potentially be due to an inner filter effect and thereby an artifact from the measurement. The lifetime of these singlet excited states was quenched to the sub‐ns regime with 0.89 ns and 0.52 ns, respectively. To characterize the triplet excited state of the porphyrins within the MOFs, the particle size had to be reduced to minimize scattering effects (see Figure S29, Supporting Information, for unresolvable kinetics from large MOF particles). A suitable method was to suspend the MOF nanoparticles in the presence of NH_2_‐terminated polyethylene glycol (PEG‐NH_2_) as described in the Supporting Information on page S25.^[^ ^24^ ^]^ The transient absorption spectra of the MOFs PCN 221 2 H and PCN 221 Zn shown in **Figure** **7**a,b exhibit a positive absorption feature emerging around 390 and 490 for the modified PCN 221 2 H, and 400 and 510 nm for PCN 221 Zn, while their ground‐state bleaching occurs at 430 and 455 nm, respectively. These bands are red‐shifted by 10 nm relative to their unmodified MOFs (see Table S7 and Figure S29, Supporting Information), indicating a potential interaction between PEG‐NH_2_ and the porphyrin within the MOF. The positive absorption features at 490 nm and 510 nm for PEG‐NH_2_‐modified PCN 221 2 H and PCN 221 Zn are characteristic of the porphyrin triplet state^[^ ^71^ ^]^ and the charge transfer band of [M‐TCPP]^−+^ cation.^[^ ^74^ ^]^ which might originate from electron transfer from the M‐TCPP to the Zr‐cluster. The kinetic fitting of the ground state bleach at 430 nm (PCN 221 2 H) and 450 nm (PCN 221 Zn), as well as the excited state absorption at 390 nm (PCN 221 2 H) and 400 nm (PCN 221 Zn), yielded, again, biexponential fittings. However, in the MOFs, the photoproducts decay with lifetimes on the order of 0.2 to 12.5 μs, which are three orders of magnitude faster than in the free porphyrin linkers, as can be compared in Table 3 (detailed datasets see Table S7, Supporting Information). The strong quenching of the singlet and triplet excited states in the MOFs compared to the free linkers might indicate photoinduced electron transfer from the M‐TCPP linkers to the Zr(IV)‐based clusters. The recombination of the charge‐separated state back to the ground state typically occurs on a relatively short timescale (≈90 ps).^[^ ^43^, ^75^ ^]^

The mixed metal MOF, PCN 221 ZnNi, showed very weak singlet state emission at 660 and 718 nm, but the lifetime was quenched below the instrumental resolution and below 1 ns. The transient absorption data did not show any long‐lived photoproduct either, indicating that additional excited state deactivation takes place in the mixed metal MOF, probably due to electron transfer between the Zn‐ and Ni‐porphyrin and additional nonradiative decays.

In addition to excited‐state electron transfer from the excited‐state linker to the Zr‐cluster, electrons can be donated from the electron donor, TEOA, to the porphyrin‐based photosensitizer. The latter was investigated via time‐resolved transient absorption spectroscopy on the most effective photocatalytic MOF, PCN 221 Zn. Specifically, the decay kinetics at 455 nm and 400 nm were probed in the absence and presence of TEOA (Figure 7c). It was found that the presence of TEOA results in a shortening of the lifetimes of both triplet state components from 0.34 and 4.62 μs to 0.19 and 3.00 μs (see Table S7, Supporting Information), supporting the hypothesis that electron transfer takes place from the electron donor to the excited state porphyrin linker.

Additionally, the photochemical behavior of PCN 221 Zn was examined via steady‐state UV–visible absorption spectroscopy under actual photocatalytic HER conditions, in the presence of H_2_O and TEOA, and upon continuous irradiation for 45 min. The sample spectra, focusing on the Q‐band region between 500 nm and 800 nm, are shown in Figure 7d. Interestingly, the Q‐band features at 520, 557, and 596 nm are qualitatively maintained, and two new bands arise at 624 and 754 nm, also signified by the experimental observation of the sample turning blue. The new spectrum can be assigned to the two‐electron reduced species, a chlorin derivative (Zn‐TCPC), and the four‐electron reduced species, a bacteriochlorin (2,3,12,13‐tetrahydroporphyrin) derivative (Zn‐TCBC), similar to the observation reported by Windle et al. when studying the Zn‐TCPP‐based molecular dyads in aq. homogenous condition.^[^ ^76^, ^77^ ^]^ Unlike in DMF, H_2_O can provide H^+^, leading to the formation of these species within the MOF environment as well. These chlorin and bacteriochlorin species might be involved in the catalytic cycle in addition to the Zr‐cluster of the SBU.

**Figure 8:**
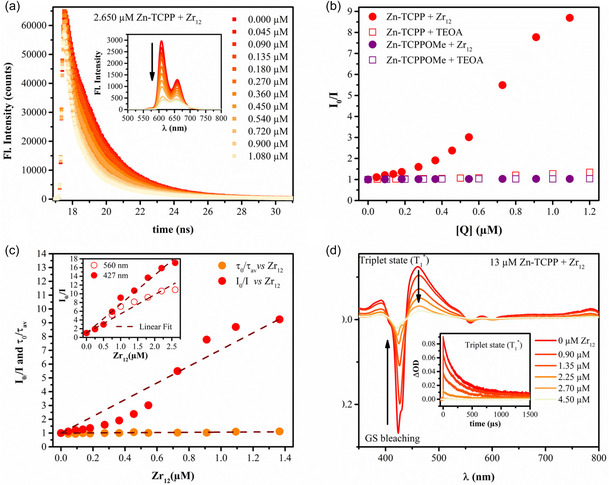
a) Fluorescence decay curves of Zn‐TCPP (≈2.65 μM) at various concentrations of the Zr_12_ cluster ([0 μM] → [1.080 μM]) under deaerated DMF at *λ*_ex_ = 372 nm. The decay curves were acquired by using a 495 nm long‐pass filter. Inset: Corresponding steady‐state fluorescence spectra at an excitation wavelength of 427 nm. b) Plots of I_0_/I for 2.65 μM Zn‐TCPP and 2.75 μM Zn‐TCPPOMe as a function of the concentration of quenchers (TEOA and molecular Zr_12_ cluster) obtained from the singlet state quenching experiments in deaerated DMF, and at *λ*_ex _= 372 nm. c) Plots of *I*_0_/*I* and τ_0_/_ττ_ for the Zn‐TCPP as a function of the concentration of Zr_12_ cluster obtained from the fluorescence quenching experiments. Inset showing plots of *I*_0_/*I* as a function of the concentration of Zr_12_ cluster obtained from the fluorescence quenching experiments under two sources of light excitation (427, and 560 nm), and d) ns‐transient absorption spectra of 13 μM Zn‐TCPP linkers in deaerated DMF at a time delay of 100 ns, and corresponding kinetic traces of the triplet state at 460 nm as a function of the concentration of Zr_12_ cluster ([0 μM] → [4.50 μM]), *λ*_ex(pump) _= 410 nm.

To test the light‐driven electron transfer from the photoexcited porphyrin to the Zr‐cluster in the absence of any 3D structural effects induced by the MOF structure, quenching studies in homogeneous conditions were conducted with the Zn‐porphyrin in DMF. To this end, the emission intensity and triplet state lifetime of Zn‐TCPP linker and its methyl ester form, Zn‐TCPPOMe, which cannot form hydrogen bonds (synthesis see Supporting Information), were measured in the presence of various concentrations of TEOA and a Zr_12_‐cluster. The Zr_12_‐cluster is an acrylate‐protected cluster that, unlike the SBU, is prevented from hydrogen bonding and cannot build up a 3D MOF structure. It was found that the excited state fluorescence intensity of Zn‐TCPP was significantly quenched by Zr_12_ (**Figure** **8**a), however, the TEOA did not yield significant quenching, and the protected Zn‐TCPPOMe was also not quenched significantly, as shown in the Stern‐Volmer plot in Figure 8b and the raw data in Figure S23a‐d, Supporting Information. In the Stern‐Volmer plot, the luminescence intensity in the absence of quencher (*I*_0_) divided by the luminescence intensity in the presence of quencher (I) is plotted against the quencher concentration ([Q]), and an increase of *I*_0_/*I* with increasing quencher ([Q] = [Zr_12_]) concentration signifies excited state deactivation, for example, via electron transfer. Quenching of Zn‐TCPP excited state may occur due to equilibrium or ground state association between chromophores or electron transfer to Zr(IV) center,^[^ ^78^ ^]^ which possibly leads to a more complex *I*_0_/*I* curve against the Zr_12_ concentration. This hypothesis is verified by a change in the absorption profile and reduction of the Soret and Q bands of Zn‐TCPP versus. [Zr_12_], shown in Figure S23b, Supporting Information, inset. Notably, distinct isosbestic points are observed, suggesting the formation of a new species. This indicates that the —COO^−^ group in Zn‐TCPP can replace an acrylate ligand on the Zr_12_ cluster.

Additionally, time‐resolved fluorescence decay curves of Zn‐TCPP at various concentrations of the Zr_12_ in deaerated DMF were recorded, see Figure 8a and Figure S24, Supporting Information, for the typical dataset for the fluorescence decay curve and the fitting. The fitting parameters obtained from the decay analysis are listed in Table S5. Upon addition of Zr_12_, two components in the decay are identified: one corresponding to unreacted Zn‐TCPP (2–2.55 ns), and the other to the associated Zn‐TCPP–Zr_12_ complex, which exhibits fluorescence with a relatively low quantum yield and a shorter lifetime (0.5–0.8 ns), very relevant to the component's lifetime in PCN 221 Zn MOF (Table 3). Therefore, the average lifetime calculated remains around 1.8–2.0 ns, remains nearly unchanged, and an almost horizontal line is obtained when $\frac{\tau_{0}}{\tau_{\text{av}}}$ is plotted against various Zr_12_ concentrations (Figure 8c) with *K*_SV_ = 6.0 × 10^4^ M^−1^, indicating a negligible dynamic quenching, while the Stern–Volmer model^[^ ^79^, ^80^, ^81^ ^]^ was applied by plotting $\frac{I_{0}}{I}$ versus Zr_12_ $\left(\frac{I_{0}}{I}=1+K_{s}\left[Zr_{12}\right]\right)$. Assuming a pure static quenching mechanism, the static quenching constant (*K*_*s*_) values were calculated to be 6.0 × 10^6^ M^−1^. The quenching has also been performed under excitation at 560 nm, which targets the Q bands. A comparison of $\frac{I_{0}}{I}$ versus. Zr_12_ is made for both in Figure 8c inset and S25–S26, Supporting Information.

The static quenching constant (*K*_s_) decreases nearly 1.5 times to 4.4 × 10^6^ M^−1^ at 560 nm excitation, indicating that each excitation wavelength can access different excited states with differing reactivity, as reported by multiple research groups.^[^ ^64^ ^]^

To elucidate the quenching of the triplet excited state of Zn‐TCPP in the presence of Zr_12,_ ns‐transient absorption spectroscopy was applied with excitation at 410 nm (Figure 8d). The kinetics of the ground state recovery and decay of the long‐lived triplet state are shown in Figure S27. The fitting parameters obtained from multiexponential decay analysis of the decay curves are listed in Table S6, Supporting Information. It is evident from the initial intensities shown in Figure 8d that with an increase in Zr_12_ content, the triplet state yield decreases, possibly because the excited state in the linked species (Zn‐TCPP‐Zr_12_) is quickly deactivated through different pathways. At lower Zr_12_ concentrations, two components are identified with a life span of 117 μs (40%) and 674 μs (60%), almost similar to the free Zn‐TCPP linker (145 μs; 40%, and 1246 μs; 60%). However, at relatively higher Zr_12_ concentrations, an additional species emerges with a relatively short lifetime of around 45 μs, likely originating from the newly formed associated species.

In summary, the quenching data, imply that excited‐state electron transfer is negligible from TEOA to excited‐state Zn‐porphyrins, and that excited‐state electron transfer is taking place from the photosensitizing linker to the Zr_12_ cluster (linker to cluster charge transfer, LCCT) only upon potential coordination of the porphyrin to the cluster via the free carboxylate.

**Figure 9:**
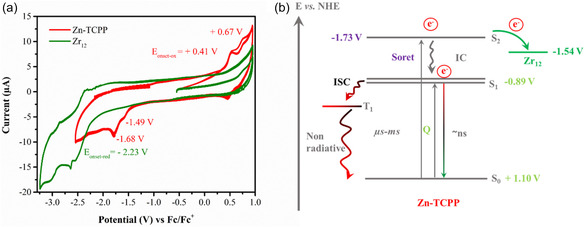
a) Cyclic voltammograms of Zn‐TCPP (red line) and Zr_12_ (green) at 100 mVs^−1^ (experimental conditions: DMF, 0.1 M nBu_4_NPF_6_ supporting electrolyte, 0.25 mM analyte, glassy carbon‐WE, Pt‐wire CE, Ag/AgCl quasi‐RE) referenced against Fc/Fc^+^. b) Schematic energy level diagram showing the electron transfer from Zn‐TCPP to the Zr_12_ cluster.

Cyclic voltammetry (CV) experiments were conducted on Zn‐TCPP and the Zr_12_ cluster in DMF using tetrabutylammonium hexafluorophosphate (nBu_4_NPF_6_) as the supporting electrolyte under an inert atmosphere. As can be seen in **Figure** **9**a, Zn‐TCPP exhibits two reversible oxidations with onset values at + 0.41 V and + 0.67 V versus. Fc/Fc^+^, along with two irreversible reductions with onset at −1.49 V and −1.68 V, centered on TCPP, while Zn remains redox‐inactive.^[^ ^82^ ^]^ The Zr_12_ cluster displays reduction onsets at more negative, higher potentials (−2.23 V *vs.* Fc/Fc^+^), corresponding to the reductions of the cluster. The respective re‐oxidation features appear as a broad signal spanning a range of potentials (from −1.7 to −3.0 V), distinctly separated from the reduction peaks. Analysis of CV (Figure 9a and S30, Supporting Information) data and steady‐state UV–vis absorption spectra (Figure 2a)^[^ ^83^, ^84^ ^]^ allowed estimation of the HOMO, first excited‐state (Q band), and second excited‐state (Soret band) potentials for Zn‐TCPP at +1.10 V, −0.89 V, and −1.73 V versus. NHE, respectively (see supporting information for calculation). The estimated reduction potential of the Zr_12_ cluster is −1.54 V versus. NHE, which is very similar to the CB potential of ZrO_2_ (−1.78 V *vs* NHE).^[^ ^85^ ^]^ Since the first excited‐state LUMO of Zn‐TCPP lies at a lower energy (−0.89 V *vs.* NHE), electron transfer from this level to the cluster is endergonic.^[^ ^86^ ^]^However, the second excited state associated with the Soret band excitation (−1.73 V *vs.* NHE) has sufficient energy to facilitate electron transfer and reduce the Zr_12_ cluster (Figure 9b), in agreement with recent findings in Zr‐based porphyrin MOFs^[^ ^64^, ^86^ ^]^ Excitation at the Soret band enables electron transfer to the cluster, reducing it to possibly Zr^+3^, which can subsequently reduce water to H_2_ (EPR spectroscopy did not provide conclusive evidence of Zr^+3^due to a weak signal under low‐resolution benchtop conditions). While electron transfer from photoexcited Zn‐TCPP to Zr‐based cluster is possible, the quantum efficiency may be low, explaining the moderate turnover number in HER.

**Scheme 2:**
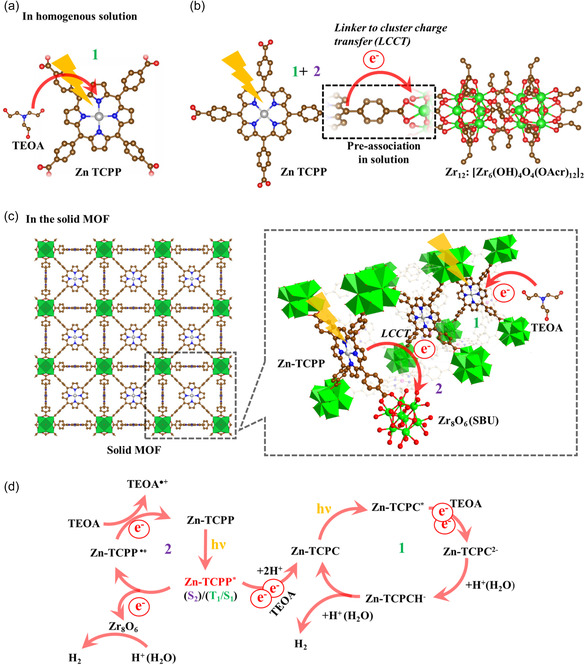
Photophysical processes that occur after the irradiation of Zn‐TCPP when it remains a) alone, b) with Zr_12_ cluster in homogenous solution, and c) in the framework of solid PCN 221 Zn that shows (1) a reductive mechanism following a photoreduction by TEOA to chlorine derivative, and (2) an oxidative mechanism by Zr_12_ cluster. d) Schematic illustration of the electron transfer and HER mechanism through this dual photocatalytic channel. Zinc chlorin sensitizer (Zn‐TCPC) can reversibly form a chlorin–phlorin anion intermediate (Zn‐TCPCH^−^), as a hydride supplying catalyst^[^ ^76^ ^]^ to H^+^, while Zr(IV) in Zr_8_O_6_ cluster, reduced after electron transfer, acts as a new site for proton reduction.

**Scheme** **2** summarizes the possible HER pathways in the representative MOF, PCN 221 Zn, including its homogenous reference experiments (homogeneous Zn‐TCPP and Zr_12_, and Zn‐TCPP alone) (Scheme 2). In Zn‐TCPP alone, there is an additional pathway (path b, see Figure S32, Supporting Information) for photocatalytic hydrogen evolution (HER) in PCN 221 Zn and Zn‐TCPP and Zr_12_. This dual‐pathway mechanism likely explains the higher TON values of PCN 221 Zn or Zn‐TCPP and Zr_12_ system relative to their molecular analogs. While in the pathways, although Zn in PCN 221 Zn and Zn‐TCPP does not directly participate in catalysis, its presence enhances TEOA coordination, promoting oxidation and oxidative quenching, increasing their activity over PCN 221 2 H and H_2_TCPP.^[^ ^87^ ^]^

In contrast, Ni(II) in PCN‐221(ZnNi), PCN‐221(Ni), and Ni‐TCPP, despite its ability to accept electrons, does not exhibit significant HER activity. This may be due to (1) a weak molar absorption coefficient, reducing light absorption efficiency, (2) the known HER catalytic activity of Ni sites under acidic conditions^[^ ^59^ ^]^, which may not be optimal in the present system. More specifically for PCN‐221(ZnNi), a decrease was observed, likely due to suboptimal structural or electronic configurations within the MOF or the incompatibility of Ni sites with the current reaction conditions.

## 3 Conclusions

This study demonstrates how a Zr(IV)‐based MOF (porous coordination network, PCN 221) comprising of a light‐harvesting M(II)‐porphyrin tetra carboxylate (M‐TCPP) linker and a Zr_8_O_6_‐based SBU can reduce water to hydrogen in presence of triethanolamine (TEOA) as a sacrificial electron donor under the irradiation of a 405 nm LED source. Among the tested MOFs, varying the metal center in the porphyrin ligands reveals a distinct trend. Under photocatalytic reaction conditions, Zn‐porphyrin exhibits superior performance compared to its 2 H, Ni, and ZnNi counterparts.

Mechanistic studies using time‐resolved and steady‐state spectroscopy revealed that photocatalytic hydrogen evolution occurs via two distinct pathways. In the first pathway, initial light‐induced electron transfer takes place from a high energy excited state of the photosensitizing Zn‐TCPP ligands to the Zr_8_O_6_‐based SBU, which serves as a catalytic site. The second pathway involves the Zn‐TCPP photosensitizer itself, which gets reduced to the chlorin and bacteriochlorin species, contributing directly to hydrogen production, similar to its behavior in homogeneous systems. This dual‐channel mechanism results in significantly higher hydrogen production in the MOF‐based system compared to the homogeneous control (linker‐only). Additionally, the enhanced photocatalytic efficiency in PCN 221 Zn compared to its other counterparts (2 H, Ni, and ZnNi) is attributed to its favorable absorption properties and excited‐state characteristics, which promote efficient charge separation, transfer, and favor coordination of TEOA. The research provides a design principle for photocatalysis systems based on earth‐abundant metals in MOFs.

## 4 Experimental Section

4.1

4.1.1

##### Synthesis of PCN 221 (M: 2 H, Zn, Ni)

PCN 221 2 H was synthesized using a reported procedure^[^ ^37^ ^]^ with minor modifications to the experimental conditions. Instead of ZrCl_4,_ here ZrOCl_2_·8H_2_O is used. ZrOCl_2_·8H_2_O (0.15 g) and BA (1.5 g) were dissolved in 30 mL of DMF in a 100 mL round‐bottom flask. The mixture was ultrasonicated for 10 min until dissolved completely. Then, 0.05 g of H_2_TCPP was added to that mixture while stirring at 300 rpm for 30 min. The resultant mixture was then placed into a Teflon‐lined autoclave and finally subjected to heat treatment at 120 °C for 24 h. After cooling, the brown‐colored solids were collected by centrifugation (15 000 rpm, 30 min) and washed with fresh DMF and acetone three times to remove the unreacted reagents. The obtained sample was dried at 50 °C for 24 h for further characterization. ATR IR (cm^−1^): 1607.7 (m), 1556.2 (m), 1508.8 (m), 1420.3 (s).

For PCN 221 Zn and PCN 221 Ni, and PCN 221 ZnNi, 0.054 g of Zn TCPP, 0.051 g Ni‐TCPP, and 0.026 g of Ni TCPP and 0.027 g of Zn TCPP (Ni: Zn = 1:1) were added, respectively, to the mixture of ZrOCl_2_·8H_2_O (0.15 g) and BA (1.5 g)). Thereafter we followed the same protocol as mentioned above for PCN 221 2 H ATR‐IR (cm^−1^): (PCN 221 Zn: 1607.7 (m), 1556.2 (m), 1508. (m), 1420.3 (s), 998 (Zn‐N), PCN 221 Ni: 1595 (m), 1537 (m), 1492. (m), 1416.3 (s), 1004.5 (Ni‐N), PCN ZnNi: 1603.6 (m), 1556.3 (m), 1506.3 (m), 1418.3 (s), 998.4 (*M*‐N, M: Zn/Ni).

##### Steady‐State and Time‐Resolved Uv–Vis Absorption and Emission Spectroscopy

Steady‐state UV–Vis absorption spectroscopy was performed on a V‐760 JASCO UV–VIS‐NIR Spectrophotometer, and steady‐state emission spectroscopy was performed on a JASCO FP‐8500 Spectrofluorometer.

Fluorescence lifetimes were recorded with a DeltaPro from Horiba Scientific using a 372 nm pulsed laser source (Class 3B laser Product, <0.5 W peak in pulsed and CW mode) and a 495 nm long‐pass filter. The Delta Pro consists of: DeltaDiode (picosecond diode controller), DeltaHub (High throughput TCSPC controller), DPS‐1 (Detector Power supply), and a PPD (picosecond photon detection module). The Instrument response function was measured with LUDOX silica nanoparticles. Data fitting was performed using Origin software.

Transient absorption experiments occurred on an LP980‐K spectrometer from Edinburgh Instruments equipped with an iCCD detector from Andor (DH320T‐25 F‐03‐812), a monochromator (STGM325‐MA), and a photomultiplier (PMT‐LP R928P). The excitation source (pump) utilizes a pulse Nd: YAG/YVO4 laser from Ekspla (NT342B‐10‐AW) equipped with a tunable OPO (410‐2600 nm) and with a repetition rate of 10 Hz. The probe light running at pulsed mode (10 Hz) was generated by a 150 W ozone‐free xenon arc lamp with Spectrometer Controller (LP1). The sample chamber was tempered to 20 °C using a built‐in thermostat. For all spectral measurements, 20 pulses were averaged, and the signal was integrated over a 100 ns timespan to yield the data shown. For kinetic data, the bandwidth and detector sensitivity of the time‐resolved setup were set, so a good signal quality was observed prior to measurements. By then, the signal of 20 pulses was averaged to improve the signal‐to‐noise ratio, and for each pulse, a gate width of 100 ns was chosen.

##### Transmission Electron Microscopy

The samples were dispersed in ethanol and, after a supersonic bath, drop‐cast onto holey carbon support grids prior to TEM experiments. TEM imaging was carried out using a ThermoFisher Talos 200X operating at 200 kV. The system was equipped with a window‐less 4‐quadrant SuperX EDX detector.

##### XPS

A commercial XPS machine from Physical Electronics (PHI 5800 ESCA) equipped with a hemispherical electron analyzer, a monochromatic Al K_α_ X‐ray source (1486.6 eV), and a flood gun to avoid charging of the sample was used for the measurements. Survey spectra were recorded using a pass energy of 93.9 eV, and detailed spectra with 58.7 eV. Both angles (angle of photon incidence on the sample and angle of emitted photoelectrons) are 45° with respect to the surface normal (sample holder, respectively). The binding energies of all spectra were calibrated with respect to the C 1s peak of ubiquitous carbon, which was fixed at a binding energy (BE) of 284.8 eV. The data were evaluated (deconvolution of spectra) by using the commercial software package CasaXPS (Casa Software Ltd., version 2.3.23PR1.0). In a first step, a Shirley background subtraction was performed.

##### Photocatalysis

MOF hybrids (1 mg) for photocatalytic hydrogen evolution were loaded in 8 ml septum‐sealed glass vials filled with 4 mL of deaerated H_2_O/TEOA (10: l) (before use, it was deoxygenated by bubbling with Ar for 1 h) in the glovebox. The sample vials were then capped and ultrasonicated for 5 min. The vials were then placed in front of a 405 nm LED source (maximum emission between 405–415 nm, 900 mW, with a current of 0.4 A and a voltage of 14 V) with magnetic stirring in an open‐source 3D‐printed photoreactor.^[^ ^76^ ^]^ The hydrogen content in the headspace of the vial was analyzed by GC after manual injection.

##### CV

CV experiments were performed on a Pine Research Wavedriver 200 electrochemical workstation equipped with a standard three‐electrode arrangement: working electrode (WE): glassy carbon electrode (d = 3.0 mm), quasi‐reference electrode (RE): Ag/AgCl, Counter electrode (CE): Pt wire. All potentials are quoted relative to the ferrocene/ferrocenium internal standard. All experiments were performed in dry dimethylformamide (DMF) using nBu_4_NPF_6_ (0.1 M) as supporting electrolyte. The solutions were purged with argon for at least 15 min to remove O_2_ and kept under a slight positive argon pressure while performing the experiments.

## Conflict of Interest

The authors declare no conflict of interest.

## Author Contributions

**Subrata Mandal**: conceptualization (equal); data curation (lead); formal analysis (lead); investigation (lead); methodology (lead); writing—original draft (lead). **Robert Leiter**: data curation (supporting); methodology (supporting). **Johannes Biskupek**: data curation (supporting); investigation (supporting); supervision (supporting); writing—original draft (supporting). **Ute Kaiser**: funding acquisition (equal); methodology (supporting); resources (supporting); supervision (supporting). **Andrea Pannwitz**: conceptualization (equal); funding acquisition (equal); resources (lead); supervision (lead); writing—review and editing (lead).

## Supporting information

Supplementary Material

## Data Availability

The data that support the findings of this study are available in the supplementary material of this article.
