# Supplementary material for: The Zr8O6 Secondary Building Unit and Porphyrin Linker Catalyze Light‐Driven H2 Evolution in Porphyrin‐Based Metal Organic Frameworks
Source: ChemSusChem. 2025 May 12;18(14):e202500372. doi: 10.1002/cssc.202500372 (PMC12270369; doi:10.1002/cssc.202500372)
Supplement: Supplementary file 1 — Supplementary Material [file CSSC-18-e202500372-s001.pdf]

Supporting Information

The Zr_8O_6 Secondary Building Unit and Porphyrin Linker Catalyze Light-Driven H_2 Evolution in Porphyrin-Based Metal Organic Frameworks

Subrata Mandal,[a] Robert Leiter,[b] Johannes Biskupek,[b] Ute Kaiser[b] and Andrea Pannwitz [a, c, d, e]

[a] Institut für Anorganische Chemie I, Universität Ulm, Albert-Einstein-Allee 11, 89081 Ulm, Germany

[b]Central Facility of Electron Microscopy, Electron Microscopy Group of Material Science, University of Ulm, Albert-Einstein-Allee 11, Ulm 89081, Germany

[c] Institut für Anorganische und Analytische Chemie, Friedrich-Schiller-Universität Jena, Humboldtstr. 8, 07743 Jena, Germany

[d] Center for Energy and Environmental Chemistry Jena (CEEC), Friedrich-Schiller-Universität Jena, Philosophenweg 7a, 07743 Jena

[e] Helmholtz Institute for Polymers in Energy Applications Jena (HIPOLE Jena), Helmholtz-Zentrum Berlin für Materialien und Energie (HZB), Lessingstraße 12–14, 07743 Jena, Germany

Table of Contents

Experimental 2
- Materials 2
- Instrumentations 2
- Synthesis of H_2TCPPOMe 4
- Synthesis of H_2TCPP 4
- Synthesis of Zn-TCPP 4
- Synthesis of Ni-TCPP 5
- Synthesis of Zn-TCPPOMe 5
- Protocol for MOF Digestion and ^{1}H NMR Analysis 13
- Synthesis of Zr_{12} clusters 16
- Stern Volmer experiments 21
- MOF sample preparation for the transient absorption measurements with better quality: 26
- Cyclic voltammetry 27
  - Energy level of HOMO and LUMO in Zn-TCPP and reduction potential of the Zr_{12} cluster (*vs* NHE) 27

References 29

Experimental

Materials

Pyrrole (reagent grade, 98%), methyl p-formyl benzoate (99%), propionic acid (≥99.5%), N,N′-dimethylformamide (≥99%), zinc(II) acetate dihydrate (≥98%), nickel(II)chloride hexahydrate ($NiCl_2{\cdot}6H_2O$) (99.9%), zirconium(IV) oxychloride octahydrate ($ZrOCl_2{\cdot}8H_2O$) (≥99.5%), benzoic acid (≥99.5%), anhydrous zinc chloride ($ZnCl_2$) (≥97%), zirconium(IV) isopropoxide isopropanol complex [$Zr(O^nPr)_4{\cdot}(CH_3)_2CHOH$] (99.9%), acrylic acid, triethanolamine (TEOA), and potassium hydroxide (KOH) were purchased from Sigma Aldrich, Merk ABCR, TCI, or Alfa Aesar. Unless otherwise stated, all solvents (99.9% purity or higher) were used without further purification.

Instrumentations

^1H nuclear magnetic resonance (NMR) spectroscopy for the synthesized M-TCPP linkers and digested MOF was performed at 298 K using a Bruker DRX 400 MHz spectrometer. Chemical shift values are reported in parts per million (ppm) and referenced to the residual peaks of the corresponding solvent. The spectra were analyzed using MestReNova software.

Infrared spectra were recorded using a Bruker Alpha II spectrometer equipped with an ATR Platinum Diamond unit. The data were collected over 24 scans at a resolution of 4 cm^{-1}.

A PANalytical X'Pert PRO diffractometer, equipped with Cu Kα radiation and a Pixel detector, was employed to acquire powder X-ray diffraction (PXRD) patterns of the MOF samples. Data were collected over a 2θ range of 5–20° at a scan rate of 0.5° min^{-1}.

High-resolution transmission electron microscopy (HRTEM) as well as Scanning transmission electron microscopy (STEM), in combination with energy-dispersive X-ray spectroscopy (EDX), was conducted on the MOF particles using a ThermoFisher Talos F200X microscope. This instrument was operated at an acceleration voltage of 80 kV and 120-200 kV, equipped with a "Super-X" windowless 4-quadrant EDX detector, and featured a dedicated high-brightness sample holder. For sample preparation, the MOF particles were first dispersed in ethanol via ultrasonication. The resulting dispersion was then drop-casted on a copper TEM support grids with holey carbon film and allowed to dry before analysis. Quantification of EDX data (mappings, spectra, elemental ratios) were determined using the Velox software package (Thermofisher Scientific company) applying background subtraction and Schreiber-Wims k-Factor quantification.

Nitrogen physisorption measurements at 77 K were carried out using a Quantachrome QuadraSorb apparatus. Prior to analysis, the MOF samples were degassed at 100°C for 24 hours. Data processing was performed using QuadraWin software.

X-ray photoelectron spectroscopy (XPS) measurements were conducted by placing the powdered samples onto a conductive Au substrate, which was then secured to the XPS sample holder. Data acquisition was performed using a UHV Multiprobe system (ScientaOmicron, Germany) equipped with a monochromatic Al Kα X-ray source and an Argus CU electron analyzer, offering an energy resolution of 0.6 eV. Charge compensation was achieved using an electron flood gun (NEK 150, Staib, Germany) operating at 6 eV and 50 µA. Background subtraction was applied, and the spectra were calibrated to the C 1s peak at 284.6 eV before fitting with Voigt functions (30:70 ratio).

Diffuse-reflectance UV-vis spectra of the MOF solids were recorded using a Shimadzu UV-2600 UV-vis spectrophotometer. The measurements were performed on samples mixed with $BaSO_4$ as a reference.

The amount of hydrogen generated was quantified using gas chromatography (GC) on a Shimadzu GC-2030 system equipped with a barrier ionization discharge (BID-2030) detector and helium as the carrier gas. The separation was performed using a Restek SH-Rt-Msieve 5A column (ID: 0.32 mm, film thickness: 30 µm, length: 30 m) with the oven temperature maintained at 80 °C. A 100 µL sample of the gas phase from the headspace of 8 mL sealed photocatalytic reaction vials (GC vials) was analyzed using a gastight syringe (Hamilton). Calibration of the GC was carried out by injecting varying known amount of standard H_2 gas.

Unless otherwise stated, all spectroscopic samples were prepared in 1 cm pathlength airtight quartz glass cuvettes in an argon-filled glovebox (MBraun) and typically handled at 20 °C (room temperature). Solvents used for spectroscopic analyses were of HPLC grade or higher and were stored under argon in the glovebox.

Steady state UV-Vis absorption spectroscopy was performed on a V-760 JASCO UV-VIS-NIR Spectrophotometer and steady state emission spectroscopy was performed on a JASCO FP-8500 Spectrofluorometer.

Fluorescence lifetimes were recorded with a DeltaPro from Horiba Scientific using a 372 nm pulsed Laser source (Class 3B Laser Product, <0.5 W peak in pulsed and CW mode) and a 495 nm long pass filter. The Delta Pro consists of: DeltaDiode (Picosecond diode controller), DeltaHub (High throughput TCSPC controller), DPS-1 (Detector Power supply) and a PPD (Picosecond photon detection module). The Instrument response function (IRF) was measured with LUDOX silica nanoparticles. Data fitting was performed using Origin software.

Transient absorption experiments occurred on an LP980-K spectrometer from Edinburgh Instruments equipped with an iCCD detector from Andor (DH320T-25F-03-812), a monochromator (STGM325-MA), and a photomultiplier (PMT-LP R928P). The excitation source (pump) utilizes a pulse Nd: YAG/YVO4 laser from Ekspla (NT342B-10-AW) equipped with a tunable OPO (410-2600 nm) and with a repetition rate of 10 Hz. The probe light running at pulsed mode (10 Hz) was generated by a 150 W ozone-free, xenon arc lamp with Spectrometer Controller (LP1). The sample chamber was tempered to 20 °C using a built-in thermostat. For all spectral measurements, 20 pulses were averaged, and the signal was integrated over a 100 ns timespan to yield the data shown. For kinetic data, the bandwidth and detector sensitivity of the time-resolved setup were set, so a good signal quality was observed prior to measurements. By then, the signal of 20 pulses was averaged to improve the signal-to-noise ratio, and for each pulse, a gate width of 100 ns was chosen.

Cyclic voltammetry (CV) experiments were performed on a Pine Research Wavedriver 200 electrochemical workstation equipped with a standard three-electrode arrangement: working electrode (WE): glassy carbon electrode (d = 3.0 mm), quasi-reference electrode (RE): Ag/AgCl, Counter electrode (CE): Pt wire. All potentials are quoted relative to the ferrocene/ferrocenium internal standard. All experiments were performed in dry dimethylformamide (DMF) using nBu_4NPF_6 (0.1 M) as supporting electrolyte. The solutions were purged with argon for at least 15 minutes to remove O_2 and kept under a slight positive argon pressure while performing the experiments.

Synthesis of H_2TCPPOMe

Following the previously described procedure,[1,2] in a 250-mL three-necked flask containing refluxed propionic acid (100 mL), pyrrole (3.10 mL, 0.044 mol) and methyl p-formyl benzoate (6.9 g, 0.042 mol) was added. The solution was refluxed for 18 hours in darkness at 140 °C. Subsequently, the reaction mixture was cooled to room temperature. The precipitates were collected by suction-filtration and washed with methanol, and ethyl acetate. The purple solid was then dissolved in dichloromethane and purified by silica gel column (200-300 mesh) using CH_2Cl_2 and ethyl acetate (v/v, 100/1) as mobile phase (1.2 g, 1.4 mmol, 12.9 % yield). ^{1}H NMR (400 MHz, $CDCl_3$): δ 8.75 (s, 8H), 8.38 (d, 8H), 8.22 (d, 8H), 4.04 (s, 12H), -2.88 (s, 2H), (UV-vis, DMF: Soret band at 419 nm (ε: 334540L mol^{-1}cm^{-1}) and four Q bands at 513 (ε: 14170 L mol^{-1}cm^{-1}), 548, 589, and 645 nm. ATR FT-IR (cm^{-1}): 1716 (s) [C=O], 1605 (s) [C=C], 1431 (s), 1401 (s), 1270 (s) [C–O], 1177 (m), 1096 (m), 1017 (s), 963 (s), 866 (s), 805(s), 760 (s), 727 (s).

Synthesis of H_2TCPP

As reported in the literature,[3] the obtained H_2TCPPOMe (0.75 g) was stirred in THF (25 mL) and MeOH (25 mL) mixed solvent, to which a solution of KOH (2.63 g, 46.95 mmol) in H_2O (25 mL) was introduced. This mixture was refluxed for 12 hours. After cooling down to room temperature, THF and MeOH were evaporated. Additional water was added to the resulting water phase and the mixture was heated until the solid was fully dissolved, then the homogeneous solution was acidified with 1M HCl until no further precipitate was detected. The violet solid was collected by filtration, washed with water, and dried in a vacuum. ^{1}H NMR (400 MHz, d^6-DMSO): δ 8.87 (s, 8H), 8.38 (q, 16H), -2.93 (s, 2H), (UV-Vis, DMF: Soret band at 419 nm (ε: 285513 L mol^{-1}cm^{-1}) and four Q bands at 514 (ε: 14563 L mol^{-1}cm^{-1}), 549, 590, and 645 nm, and and ATR FT-IR (cm^{-1}): 1682(s) [C═O],, 1603 (s) [C=C], 1564 (m), 1400 (s), 1311 (m) [P ring], 1266 (s) [C–O], 1233 (s), 1175 (m), 1097 (m), 1017 (s), 865 (m), 792 (s), 768 (s), 724 (m) cm^{-1}.

Synthesis of Zn-TCPP

Following the previously described procedure,[4] H_2TCPP ligand (0.166 mmol, 130 mg) was dissolved in N, N′-dimethylformamide (40 mL) with zinc (II) acetate dihydrate (0.91 mmol, 200 mg). Then the mixture was heated at 120 °C for 4.5 h under an Ar atmosphere. The volume of the DMF after the reaction was reduced by distillation and the complex was precipitated by adding H_2O. The precipitate was dissolved in 0.1 M NaOH and reprecipitated by adding 1 M HCl. The solid Zn-TCPP obtained was filtered and dried at room temperature. ^{1}H NMR (400 MHz, d^6-DMSO): δ- 8.80 (s, 8H), 8.37 (8H, d), 8.31 (8H, d), UV–vis, DMF: Soret band at 427 nm (ε: 311785 L mol^{-1}cm^{-1}) and four Q bands at 518, 559 (ε: 13397 L mol^{-1}cm^{-1}), and 599 nm, and ATR FT-IR (cm^{-1}): 1682 (s) [C═O], 1601 (s) [C=C], 1564 (m), 1402 (s), 1311 (m) [P ring], 1264 (s) [C–O], 1233 (s), 1204 (s), 1173 (m), 1099 (m), 994 (s) [Zn-N], 865 (m), 792 (s), 766 (s), 716 (m).

Synthesis of Ni-TCPP

Ni-TCPP was prepared by refluxing H_2TCPP (0.8 g) in 100 mL DMF with an excess of $NiCl_2·6H_2O$ (2.5 g) for 6 h.[2]The crimson product was precipitated out by addition of water then filtered, washed with water, and allowed to dry: ^{1}H NMR (400 MHz, d6-DMSO) δ –8.16 (d, 8H), 8.33 (d, 8H), 8.79 ppm (s, 8H). UV–vis, DMF: 415 nm (ε: 41910 L mol^{-1}cm^{-1}), 527 nm (ε: 3113 L mol^{-1}cm^{-1}), and ATR FT-IR (cm^{-1}): 1690 (s) [C═O], 1605 (s) [C=C], 1568 (m), 1403 (s), 1313 (m) [P ring], 1274 (s) [C–O], 1230 (s), 1205 (s), 1175 (m), 1080 (m), 1002 (s) [Ni-N], 866 (m), 796 (s), 766 (s), 714 (m).

Synthesis of Zn-TCPPOMe

Following the previously described procedure,[1] a solution of TCPPOMe 0.1708 g (0.2 mmol) and $ZnCl_2$ (0.35 g, 2.56 mmol) in 30 mL of DMF was refluxed for 6h After the mixture was cooled to room temperature, 45 mL of H_2O was added. The resultant precipitate was filtered and washed with 20 mL of H_2O twice. The obtained solid was dissolved in $CHCl_3$, followed by washing three times with water. The organic layer was dried over anhydrous magnesium sulfate and evaporated to afford quantitative violet crystals. ^{1}H NMR (400 MHz, d^6-DMSO) δ –4.05 (s, 12H), 8.34 (d, 8H), 8.39 (d, 8H), 8.79 ppm (s, 8H). UV–vis, DMF: 427 nm (ε: 373569 L mol^{-1}cm^{-1}), 519, 559 (ε:14010 L mol^{-1}cm^{-1}), 599 nm, and ATR FT-IR (cm^{-1}): 1699 (s) [C=O], 1603 (s) [C=C], 1430 (s), 1268 (s) [C–O], 1231 (s), 1110 (m), 1060 (m), 996 (s), 953 (s), 862 (s), 815(s), 791 (s), 761 (s), 715 (s).

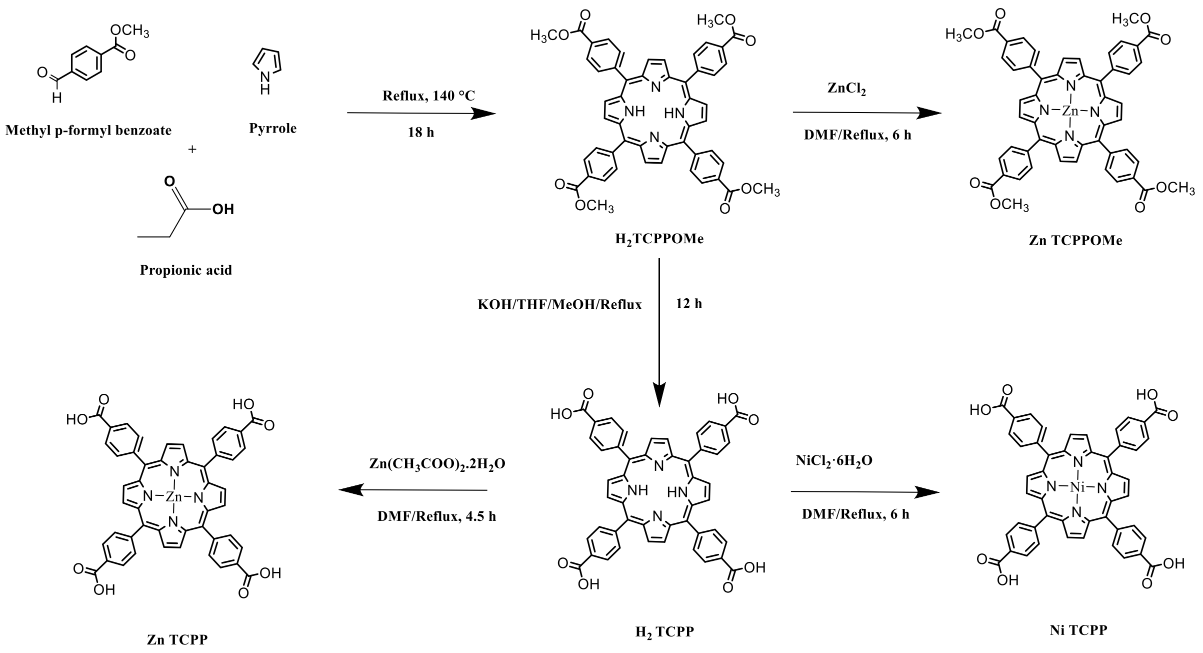

Scheme S1. Synthetic route for various M-TCPP linkers (M: H_2, Zn, and Ni) and Zn-TCPPOMe.

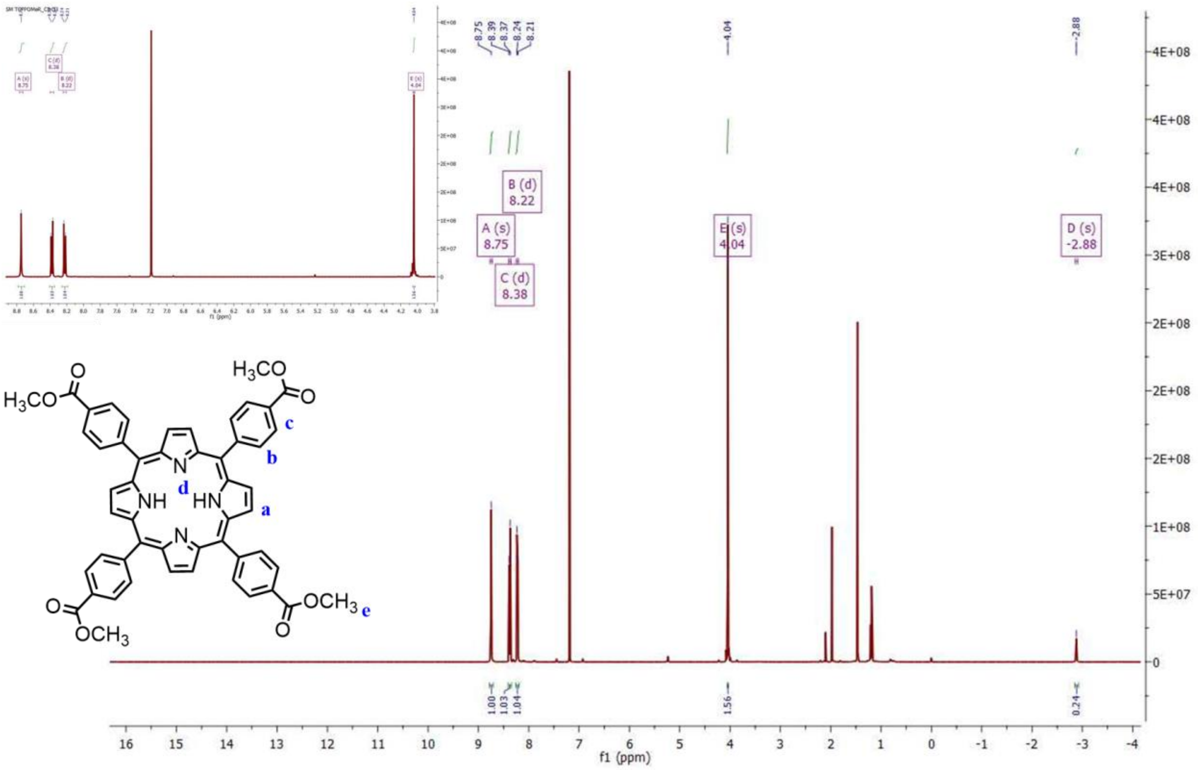

Figure S1. ^{1}H NMR spectra of H_2TCPPOMe in $CDCl_3$

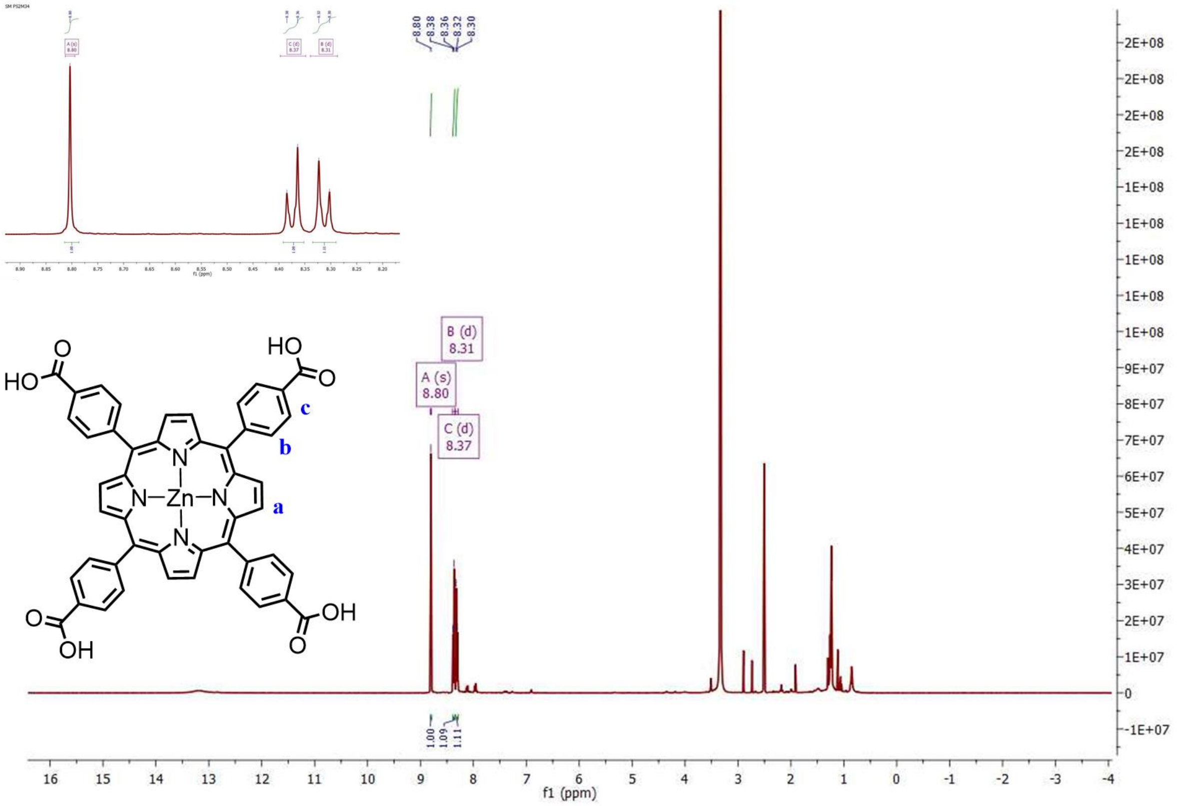

Figure S2. ^{1}H NMR spectra of Zn-TCPP in d^6-DMSO.

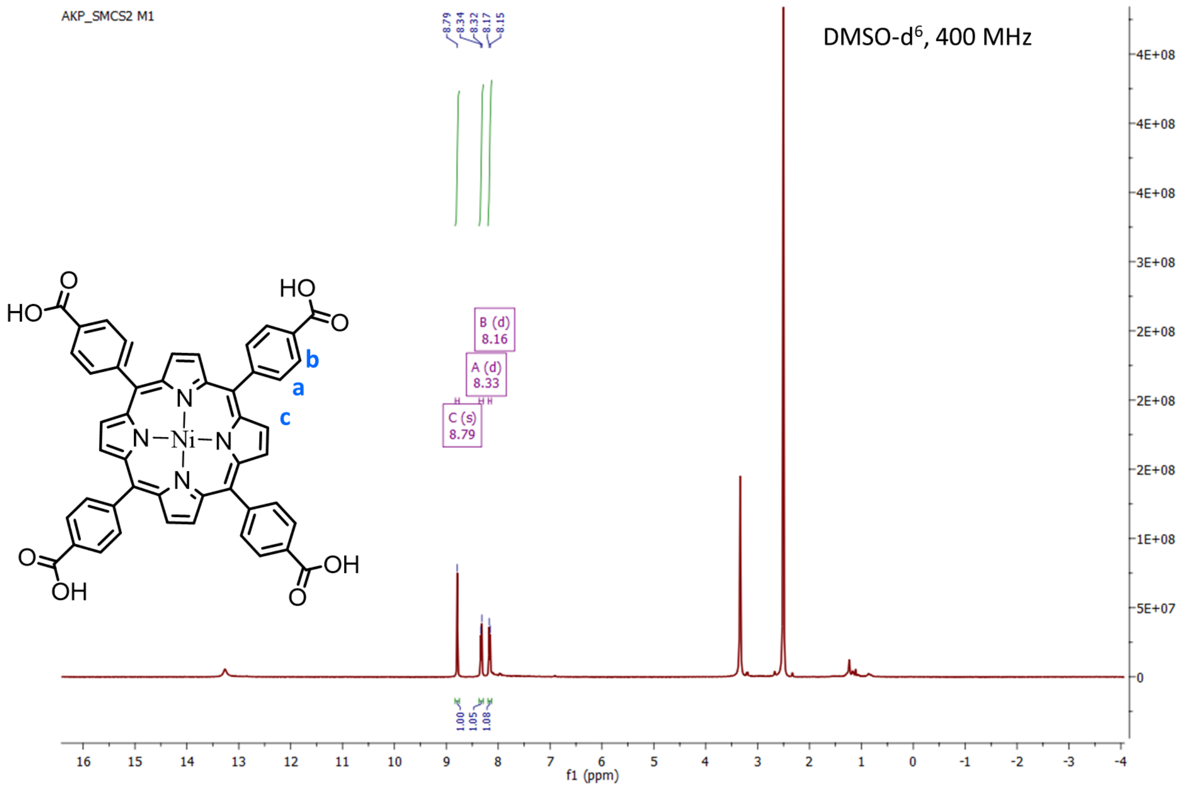

Figure S3. ^1H NMR spectra of Ni-TCPP in d^6-DMSO.

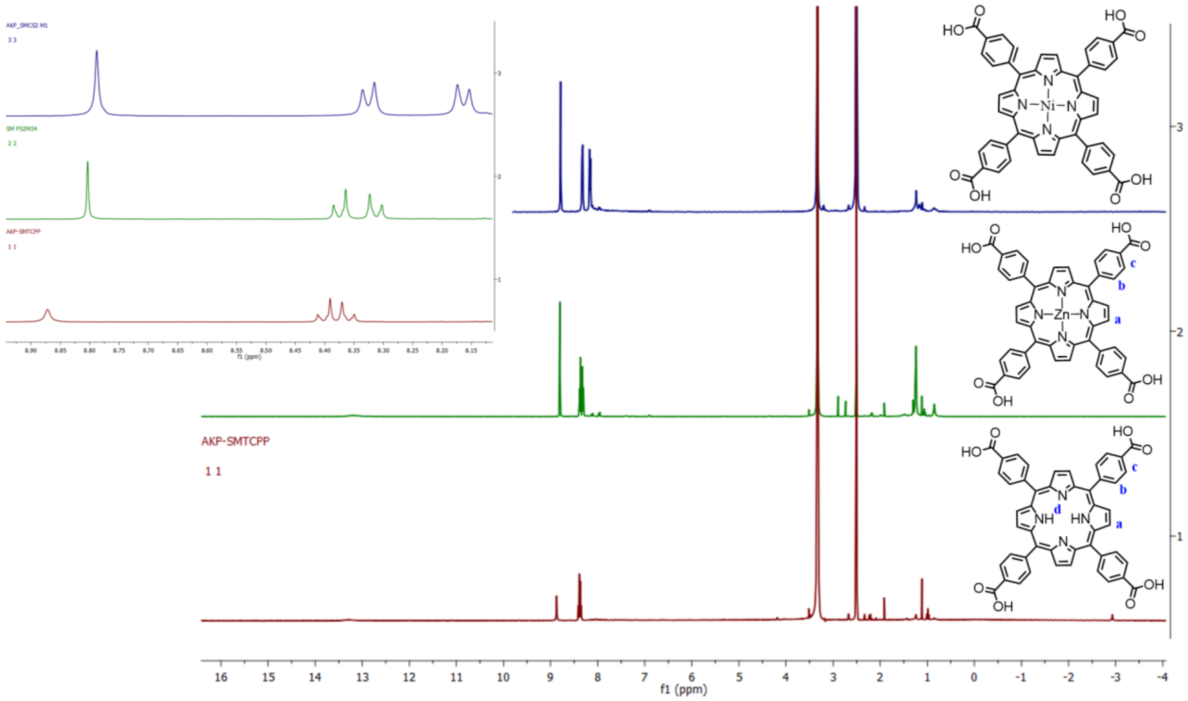

Figure S4. A comparison of ^1H NMR spectra of M-TCPP in d^6-DMSO, where M: H_2, Zn and Ni.

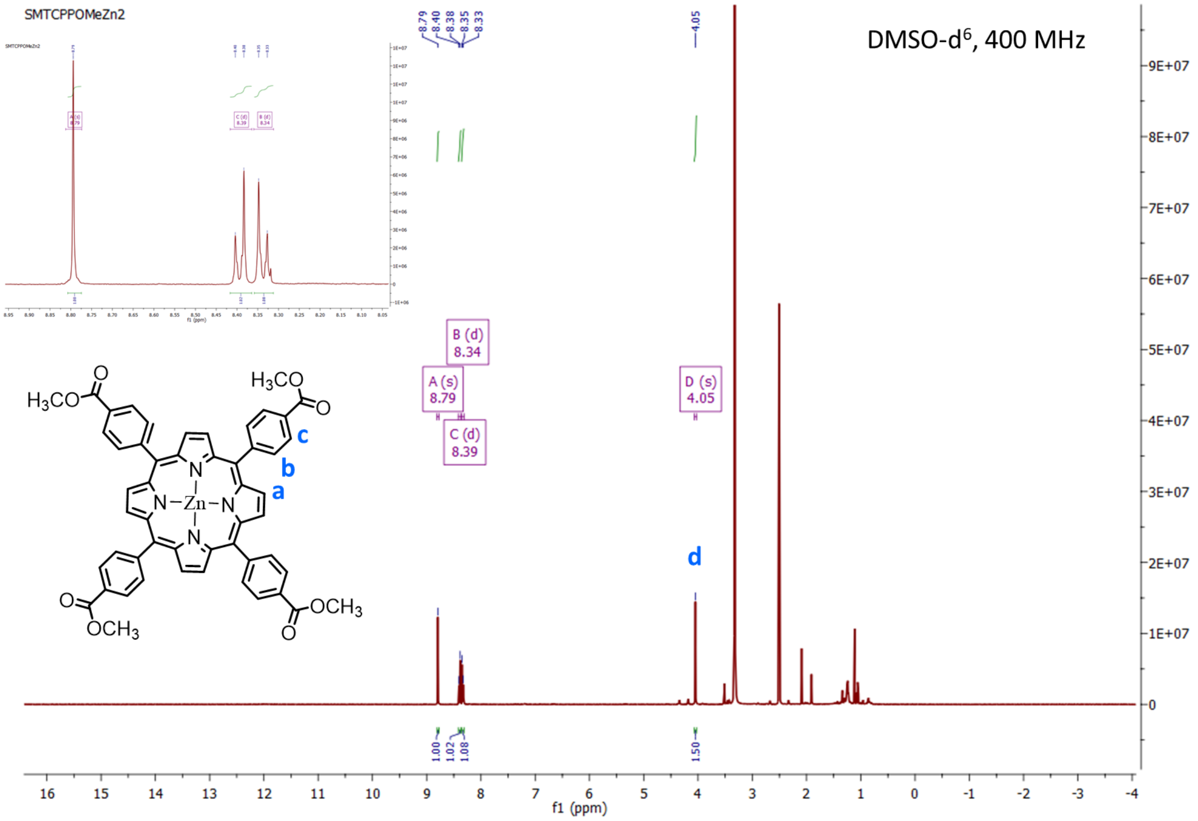

Figure S5. ^{1}H NMR spectra of Zn-TCPPOMe in d^6-DMSO.

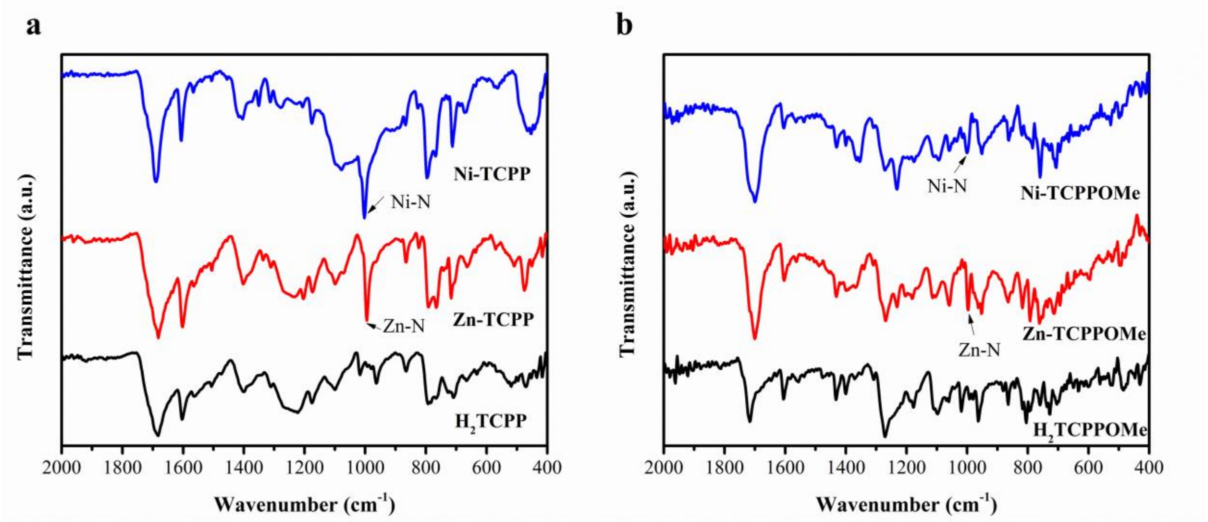

Figure S6. ATR-IR spectra of synthesized (a) M-TCPP linkers and (b) M-TCPPOMe, M: H_2 (black), Zn (red) and Ni (blue).

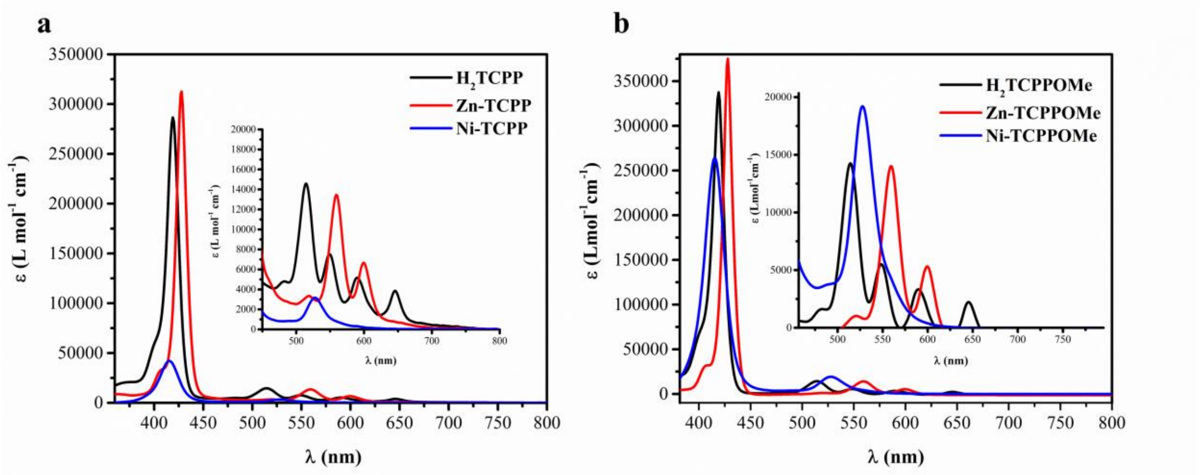

Figure S7. A comparison of steady state UV-vis spectra of M-TCPP and M-TCPPOMe in DMF solution, where M: H_2 (black), Zn (red), and Ni (blue).

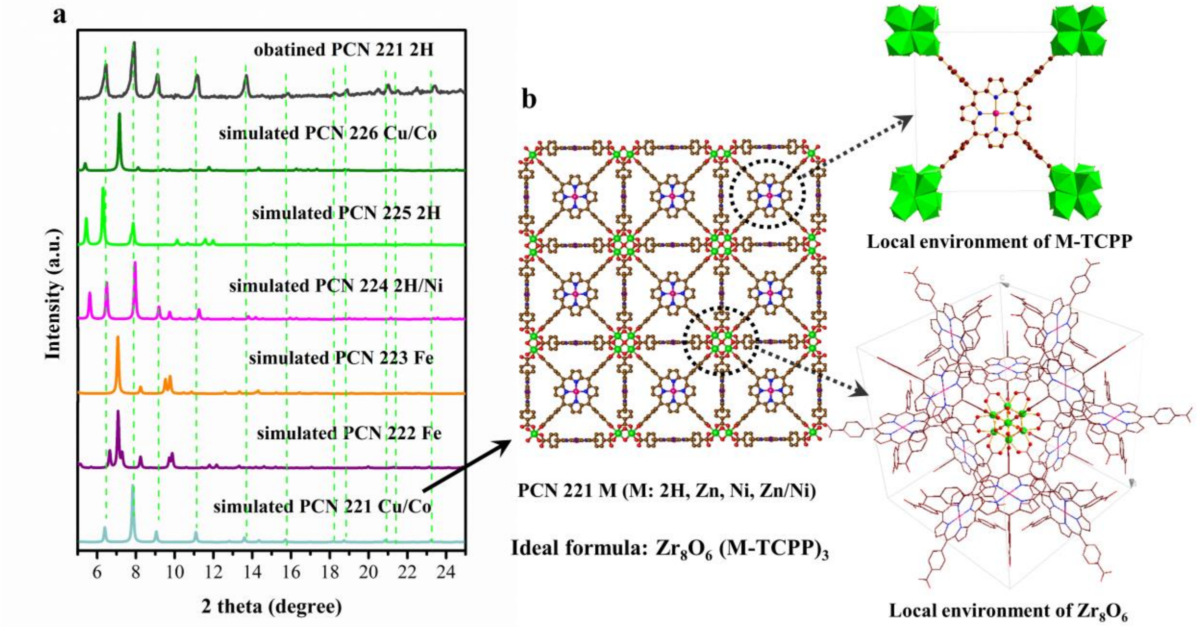

Figure S8. (a) A comparison of powder XRD data of our obtained PCN 221 2H MOF (black) with the reported PCN-based MOF series. (b) The periodic structure (2D view) of the PCN 221 showing the local environment (right) of M-TCPP (top) and the $[Zr_8O_6\ (CO_2)_{12}]^{n+}$ cluster (bottom) which has Zr_8O_6 as a core and connected to twelve M-TCPP linkers whereas each M-TCPP linker is surrounded by four Zr_8O_6 and the peripheral benzene ring remained 90 degree to the porphyrin plane. The O and carboxylate O, Zr, C, N, and M atoms are shown in red, green, brown, blue, and pink respectively. Each $[Zr_8O_6(CO_2)_{12}]^{n+}$ cluster at the top is represented by polyhedra (green) and the wireframe model at the bottom was used to show the M-TCPP, and Zr_8 environment, respectively. H atoms are omitted to get more clarity. The structure depicted here was obtained from CCDC 925058 [5] and was analyzed, and visualized using Dimond and Vesta software, respectively for enhanced clarity and precision.

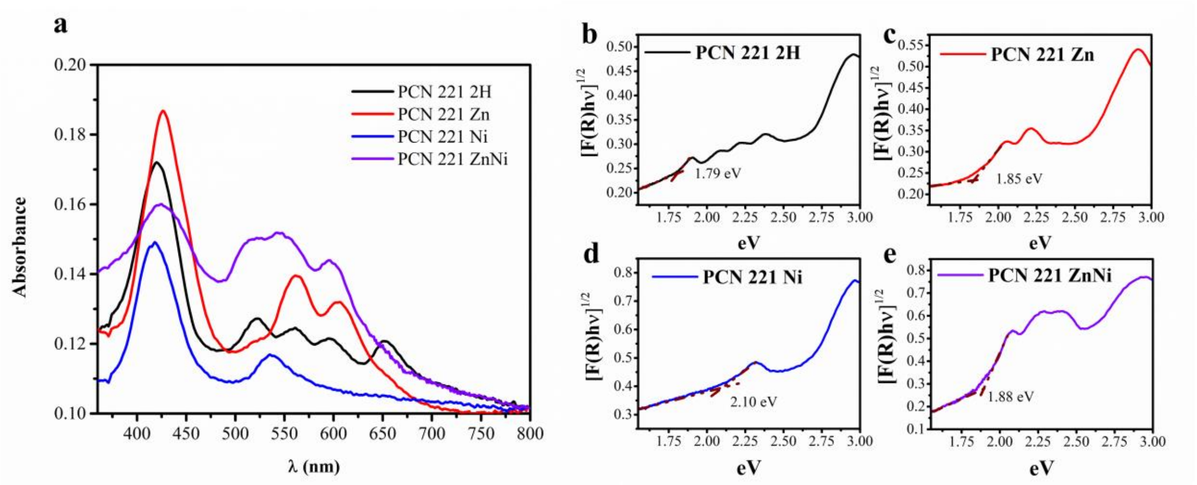

Figure S9. (a) UV-Vis absorption spectra acquired from the diffuse reflectance spectroscopy by applying the Kubelka-Munk function $F(R_\infty) = (1 - R_\infty)^2/2R_\infty$ (where $F(R_\infty)$is reflectance of an infinitely thick specimen) of the solid MOFs (a) and corresponding Tauc plots of (b) PCN 221 2H, (c) PCN 221 Zn, (d) PCN 221 Ni, and (e) PCN 221 ZnNi.

Table S1. Table of BET surface area and pore parameters obtained from N_2-sorption measurements.

**Samples**	**BET Surface area (m^2g^{-1})**	**Total pore volume (cm^3g^{-1})**		**Avg. pore diamter (nm)**	
		**DFT**	**BJH**	**DFT**	**BJH**
PCN 221 2H	1493.7	1.044	0.638	1.631	3.278
PCN 221 Zn	348.2	0.671	0.629	2.027	3.655
PCN 221 Ni	578.5	0.465	0.303	2.027	3.885
PCN 221 ZnNi	236.1	0.139	0.037	3.537	3.136

The BJH (Barrett, Joyner, Halenda) method and the modern DFT (Density Functional Theory) method are employed to ascertain pore volume and pore size distribution.

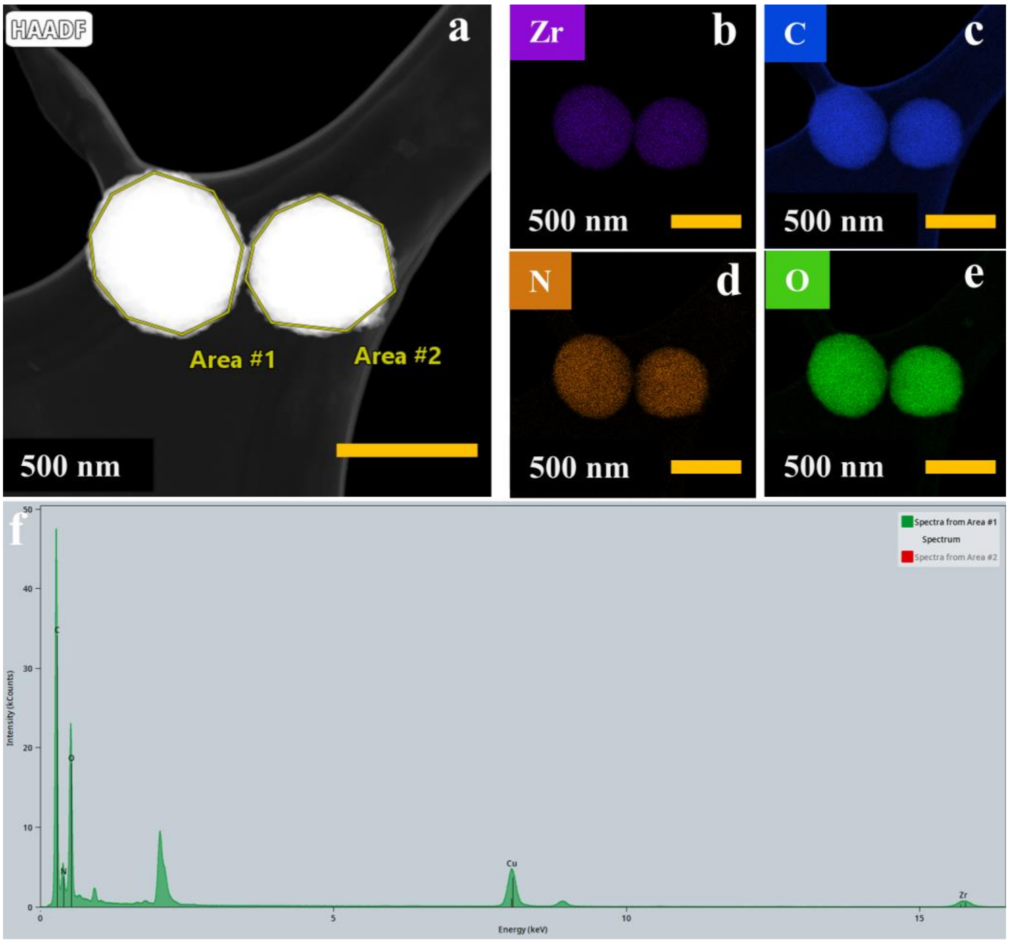

Figure S10. (a) High-angle annular dark field scanning transmission electron microscopic (HAADF-STEM) image with corresponding (b-e) EDX elemental mapping images and an integrated (f) EDX spectrum of PCN 221 2H sample. The violet, blue, orange, and green signals in (b), (c), (d), and (e) represent Zr, C, N, and O, respectively.

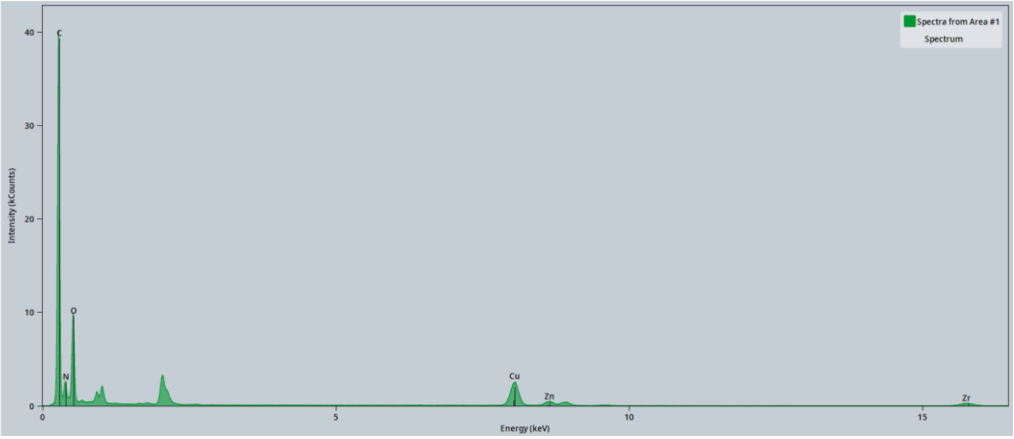

Figure S11. EDX spectrum of the PCN 221 Zn sample obtained from the HAADF-STEM image presented in **Figure 3** of the main manuscript.

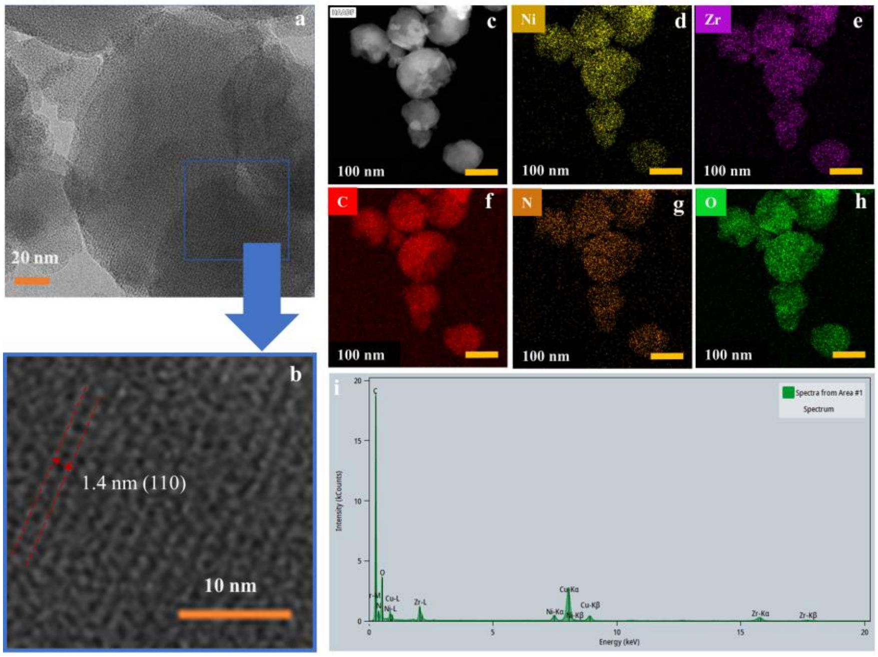

Figure S12. (a) HRTEM image of the PCN 221 Ni with higher magnification (b) showing lattice fringes corresponding to the (110) plane of cubic phase PCN 221 Ni. (c) HAADF-STEM image with corresponding (d-h) EDX elemental mapping images and an integrated (i) EDX spectrum of PCN 221 Ni sample. The yellow, violet, red, orange, and green signals in (d), (e), (f), (g) and (h) represent Ni, Zr, C, N, and O, respectively.

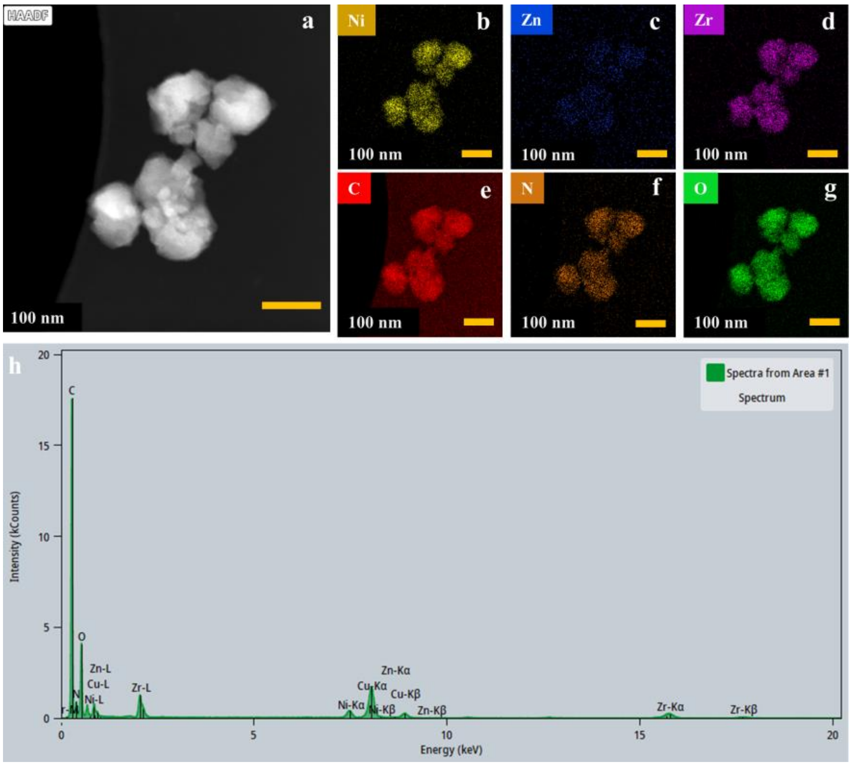

Figure S13. (a) HAADF-STEM image with corresponding (b-g) EDX elemental mapping images and integrated (h) EDX spectrum of PCN 221 ZnNi sample. The yellow, blue, violet, red, orange, and green signals in (b), (c), (d), (e), (f), and (g) represent Ni, Zn, Zr, C, N and O, respectively.

Table S2. Elemental composition of the MOFs obtained from the EDX data in **Figure S10-S13** (atomic%).

**Samples**	**Atomic %**					**Ratio obtained EDX**		**Expected ratio**[a]	
	**Zr**	**C**	**N**	**O**	**M**	**Zr/N**	**Zr/M**	**Zr/N**	**Zr/M**
PCN 221 2H	4.3 ± 0.6	69.0 ± 3.0	6.2 ± 1.2	20.5 ± 3.3	-	0.69	-		-
PCN 221 Zn	2.6 ± 0.3	76.9 ± 2.4	4.6 ± 0.9	14.9 ± 2.5	1.0 ± 0.1	0.57	2.71	0.67	
PCN 221 Ni	4.3 ± 0.5	78.4 ± 2.0	3.5 ± 0.7	12.1 ± 2.1	1.7 ± 0.2	1.22	2.52		2.67
PCN 221 ZnNi	4.9 ± 0.6	77.6 ± 2.0	3.2 ± 0.6	12.3 ± 2.2	0.2 (Zn) 1.8±0.3(Ni)	1.53	2.45 (Zn+Ni)		

[a] Expected Zr/N and Zr/M atomic ratio was calculated from the general ideal formula of PCN 221 M, which is $[Zr_8O_6(M\text{-}TCPP)]_3$ or $C_{144}H_{72}M_3N_{12}O_{30}Zr_8$, where M: 2H, Zn, Ni, and 1:1 ZnNi. The quantitative composition of C and O in the table is not accurate as the carbon tape was employed as background.

Protocol for MOF Digestion and ^{1}H NMR Analysis

To prepare the samples, 1 mg of activated MOF was placed in a 4 mL glass vial. Subsequently, under air, 10 μL of DCl/D_2O and 590 μL of dimethyl sulfoxide-d_6 were added to the vial. The vials were sealed, sonicated for 15 minutes, and then left at room temperature overnight to produce a uniform, dark green solution. These solutions were then transferred into NMR tubes, and **^{1}H NMR** spectra were recorded using a Bruker DRX 400 MHz spectrometer (**Figure S14**). The spectra were referenced to tetramethylsilane, using the residual dimethyl sulfoxide-d^5 signal at δ = 2.50, and normalized to the intensities of the H_6TCPP^{2+} (corresponds to the M-TCPP in actual PCN 221 M MOF) resonances observed between 8.5 and 9 ppm ((δ 8.77 (d, J = 7.9 Hz, 8 phenyl ArH per porphyrin), 8.64 (s, 8 porphyrin ArH per porphyrin), 8.58 (d, J = 7.9 Hz, 8 phenyl ArH per porphyrin)). The peaks between 7.3 and 8.1 ppm are due to benzoate (BA) ((δ 7.86 (d, 2H), δ 7.56 (t, 1H), and 7.44 (t, 2H)), HCOOH ((δ 8.10 (s, H)), and DMF ((δ 7.96 (s, H)) that were also present in as-synthesized PCN-221M.

The actual experimental molar ratio of the [BA], [HCOOH] and [DMF] to [M-TCPP] (M = H_2, Zn, Ni, and Zn-Ni) in PCN 221 M MOF was determined from **equation S1**

$$\frac{[BA] or\ [HCOOH] or\ [DMF]}{[M\text{-}TCPP]} = \frac{\int BA\ or\ HCOOH\ or\ DMF}{n_H\ (BA\ or\ HCOOH\ or\ DMF)} \times \frac{n_H\ (M\text{-}TCPP)}{\int M\text{-}TCPP} = \frac{y}{x} \text{ or } \frac{z}{x}\ or\ \frac{n}{x} \quad \text{(S1)}$$

, where $\int BA$ or HCOOH or DMF is the integration of the BA or HCOOH or DMF at 7.86, 8.10, and 7.96 ppm, respectively, n_H (BA or HCOOH or DMF) is the number of protons corresponding to the doublet (2H), singlet (1H), and singlet (1H) respectively, n_H ($M\text{-}TCPP$) is the number of protons corresponding to the selected $[H_6TCPP]^{2+}$ at 8.77 ppm, and $\int M\text{-}TCPP$is the integration of that $[H_6TCPP]^{2+}$ doublets (8H).

The ideal chemical formula of the PCN 221 M MOF is $[Zr_8O_6\ (M\text{-}TCPP)]_3$. Charge of the SBU: $(Zr_8O_6)^{12+}$, total charge of the 3 equiv. linker $(M\text{-}TCPP)^{4-}$ is 12-. The obtained chemical formula from the experiments will be $[Zr_8O_6(M\text{-}TCPP)_x\,(BA)_y\,(HCOOH)_z]\cdot n$ DMF. While the

SBU $(Zr_8O_6)^{12+}$ connects with x equiv. $(M\text{-}TCPP)^{4-}$, y equiv. of $(BA)^-$, and z equiv. of $HCOO^-$. Based on the total charge,

$$y + z + 4x = 12 \quad \text{(S2)}$$

Therefore, using the molar ratio obtained from equation S1, and by solving the equation from **equation S2**, x and y, and z can be calculated. The DMF (n) can be directly calculated from the ratio as obtained from the equation S1, once the [M-TCPP] is calculated.

Table S3. Quantification of [M-TCPP] to benzoate and DMF in digestates of MOFs by ^{1}H NMR.

Samples	Relative area ^{1}H NMR				Molar ratio [BA]: [M-TCPP]: [HCOOH]: DMF	Calculated formula of the MOF
	**M-TCPP** δ 8.77 (8H)	**BA** δ 7.86 (2H)	**HCOOH** δ 8.10 (1H)	**DMF** δ 7.96 (1H)		$[Zr_8O_6(M\text{-}TCPP)_x(BA)_y(HCOO)_z]\cdot n$ DMF
PCN 221 H_2	1	0.55	0.10	0.02	2.2: 1.0: 0.8: 0.16	$[Zr_8O_6(H_2TCPP)_{1.71}(BA)_{3.76}(HCOO)_{1.40}]\cdot 0.27$ DMF
PCN 221 Zn	1	0.78	0.10	0.02	3.12:1.0:0.8 0.16	$[Zr_8O_6(Zn\text{-}TCPP)_{1.51}(BA)_{4.73}(HCOO)_{1.23}]\cdot 0.24$ DMF
PCN 221 Ni	1	0.73	0.06	0.06	2.92: 1.0: 0.48 0.48	$[Zr_8O_6(Ni\text{-}TCPP)_{1.62}(BA)_{4.74}(HCOO)_{0.78}]\cdot 0.78$ DMF
PCN 221 ZnNi	1	0.45	0.02	0.03	1.8:1.0: 0.08 0.24	$[Zr_8O_6(Zn/Ni\ TCPP)_{2.04}(BA)_{3.67}(HCOO)_{0.16}]\cdot 0.49$ DMF or $[Zr_8O_6(Zn\text{-}TCPP)_{0.24}(Ni\text{-}TCPP)_{1.80}(BA)_{3.67}(HCOO)_{0.16}]\cdot 0.49$ DMF [a]

[a] The ratio of Zn and Ni in PCN 221 ZnNi MOF is considered from the EDX data as listed in **Table S2**.

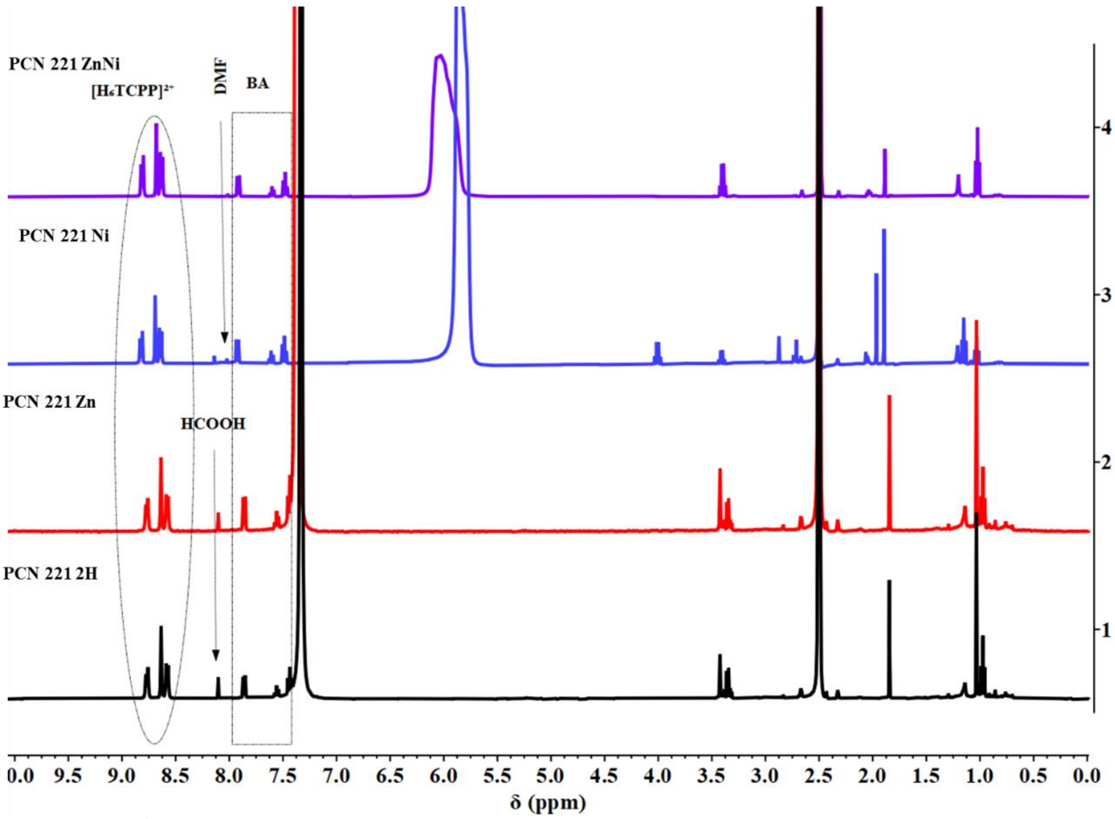

Figure S14. ^{1}H NMR spectrum of 1 mg of digested MOF in 10 μL of DCl/D_2O and 590 μL of dimethyl sulfoxide-d[6]. Black spectrum: PCN 221 2H, red spectrum: PCN 221 Zn, blue spectrum: PCN 221 Ni, and violet spectrum: PCN 221 ZnNi.

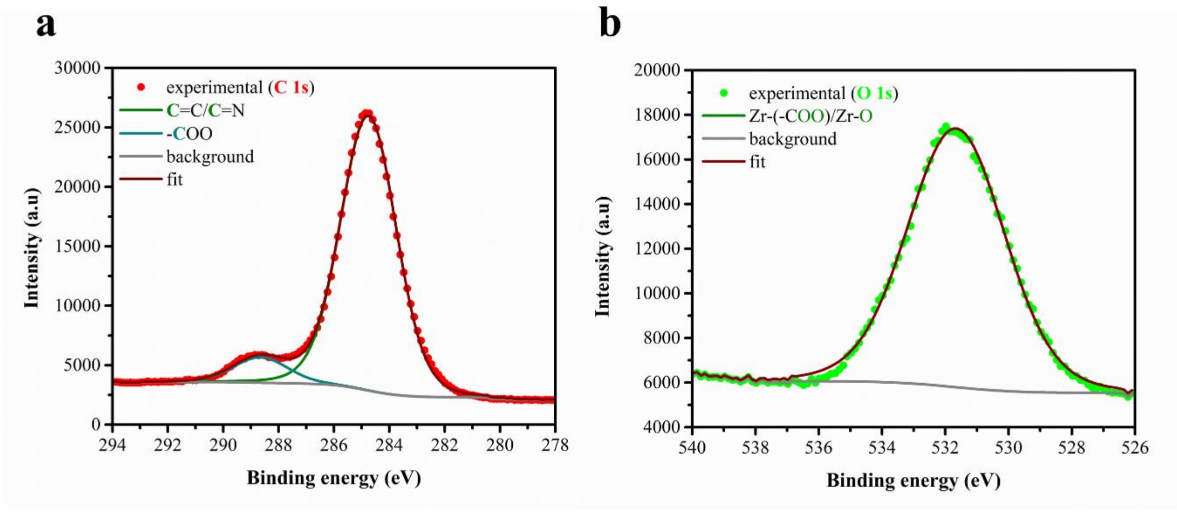

Figure S15. XPS: (a) C1s, and (b) O1s spectra of PCN 221 Zn MOF.

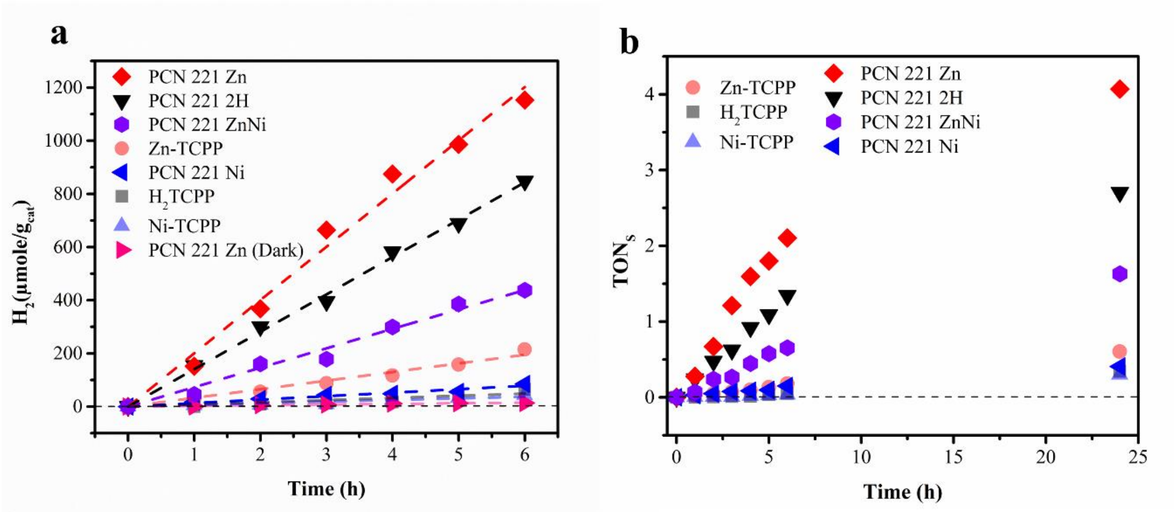

Figure S16. (a) Linear fitting (y = a + bx, a = 0) of the kinetic curves for photocatalytic HER (6h) for the PCN 221 MOFs and corresponding homogeneous M-TCPP linkers. (b) A comparison of H_2 production TON_S *vs*. time up to 24 h irradiation between PCN 221 M MOFs and their homogenous linkers. Reaction conditions: ~1 mg of MOF or ~0.75-0.90 mg of M-TCPP linkers as a photocatalyst, Solvent: deaerated H_2O: TEOA (4:1 v/v), λ: 405 nm LED source.

Synthesis of Zr_{12} clusters

The synthesis of Zr_{12}, with the chemical formula $[Zr_6(OH)_4O_4(OAcr)_{12}]_2 \cdot 6AcrOH$ (where Oacr denotes acrylate), was conducted as previously reported.[6,7] In a Schlenk tube under an argon environment, $Zr(O^nPr)_4$ (2.00 mL of a 70% weight/volume solution in n-propanol, 4.46 mmol) was combined with acrylic acid (2.00 mL, 29.2 mmol). Following homogenization for several minutes, the mixture was maintained at ambient temperature. A white solid precipitate was formed after 3 days and subsequently collected using vacuum filtration. The product was washed with 35 mL of CH_2Cl_2 and subsequently dried at decreased pressure, initially using a rotary evaporator and then under high vacuum for 6 hours. Yield: 0.44 g, 34% relative to Zr. The analysis by ^{1}H NMR spectroscopy corroborated previously reported for similar substances, hence affirming the identity of the acquired cluster. Despite extensive washing with CH_2Cl_2,, 4 to 6 molecules of 'free' acrylic acid (probably linked to the cluster via hydrogen bonding interactions) were consistently detected by ^{1}H NMR.

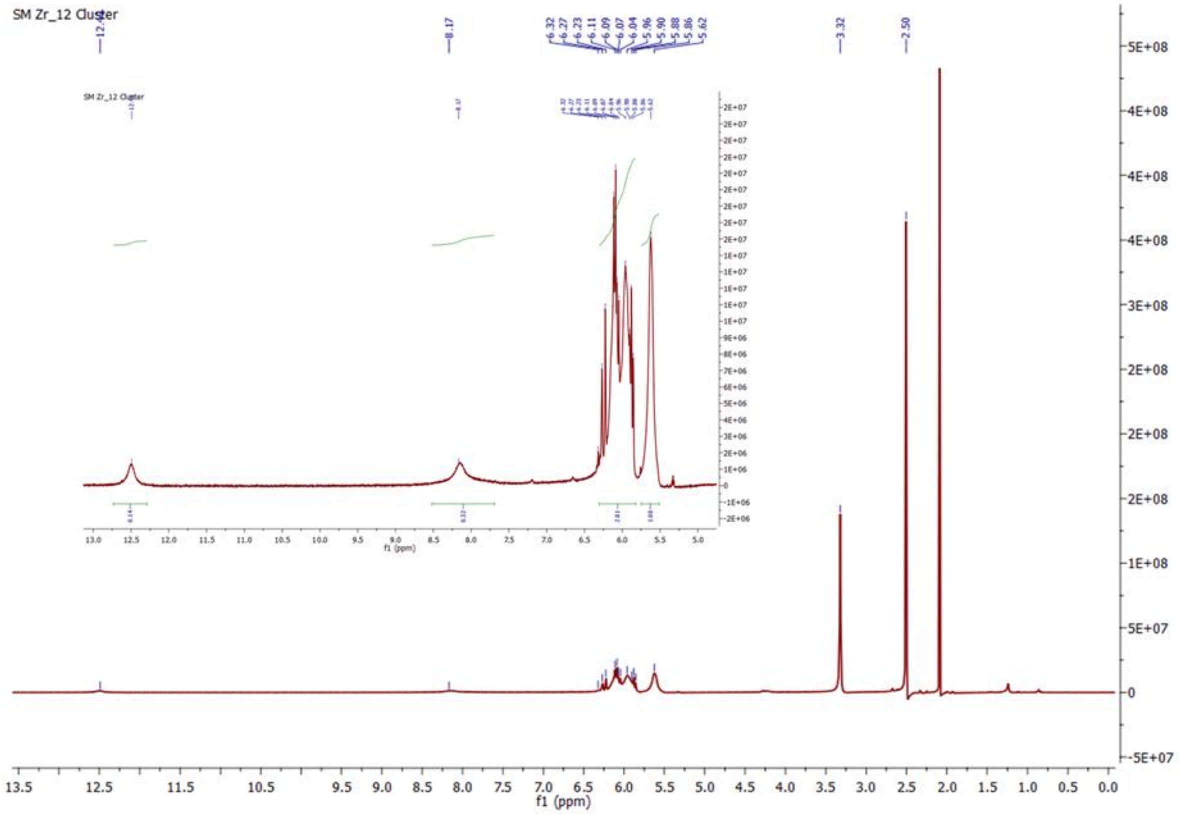

Figure S17. ^{1}H NMR spectra of $Zr_6(OH)_4O_4(OAcr)_{12}]_2{\cdot}6AcrOH$ (OAcr = acrylate), Zr_{12} cluster in d^6-DMSO.

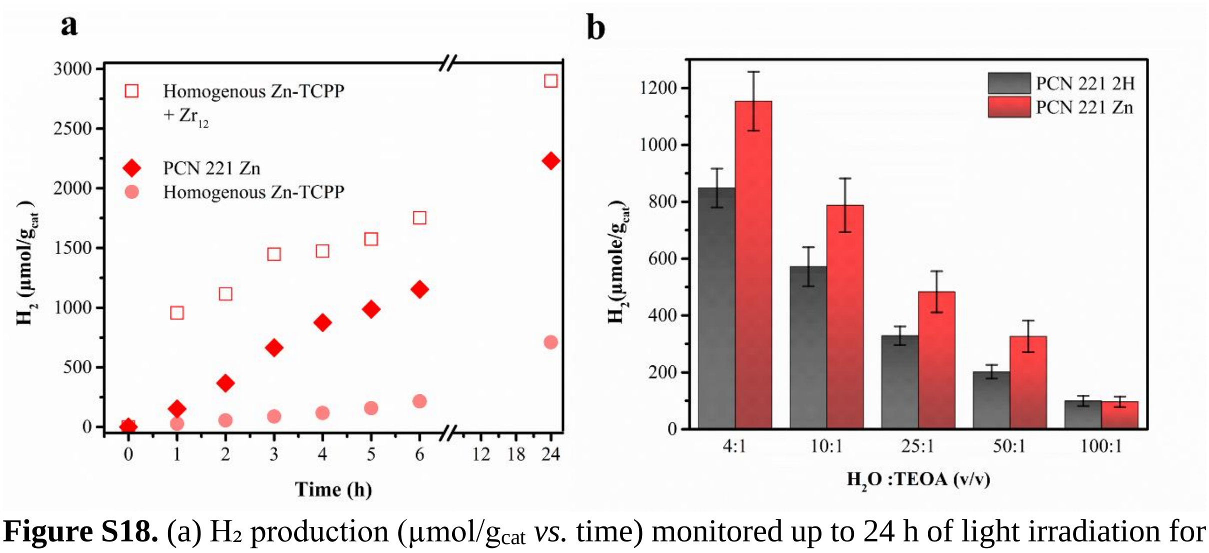

Figure S18. (a) H_2 production (μmol/g_{cat} *vs.* time) monitored up to 24 h of light irradiation for (1) Zn-TCPP alone, [1.04 μmol], (2) Zn-TCPP [0.44 μmol] + Zr_{12} [3.40 μmol], and (3) PCN 221 Zn MOF under the following reaction conditions: deaerated H_2O: TEOA (4:1 v/v); λ: 405 nm LED source. (b) Under similar conditions, the variation in H_2 production efficiency (μmole/g_{cat}) for PCN 221 Zn and PCN 221 2H was assessed in different water-to-sacrificial donor ratios after 6 hours of light irradiation.

.

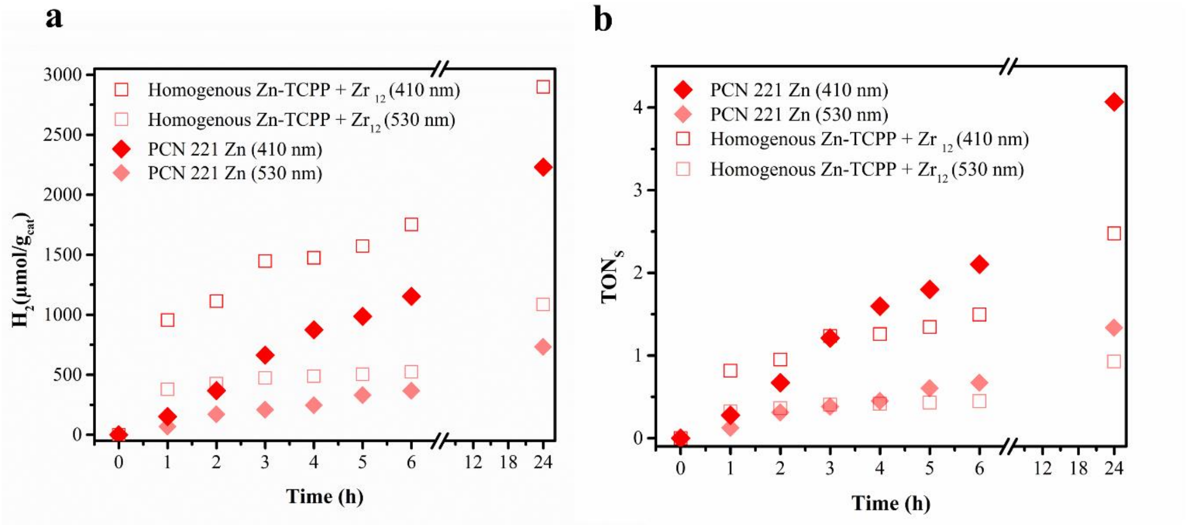

Figure S19. Comparison of H_2 production (a) μmol/g_{cat} *vs*. time and (b) TON_S *vs*. time for the (1) Zn-TCPP [0.44 μmol] + Zr_{12} [3.40 μmol], and (2) PCN 221 Zn MOF under two different sources of excitation wavelengths, 405 nm and 530 nm, solvent and donors: deaerated H_2O: TEOA (4:1 v/v).

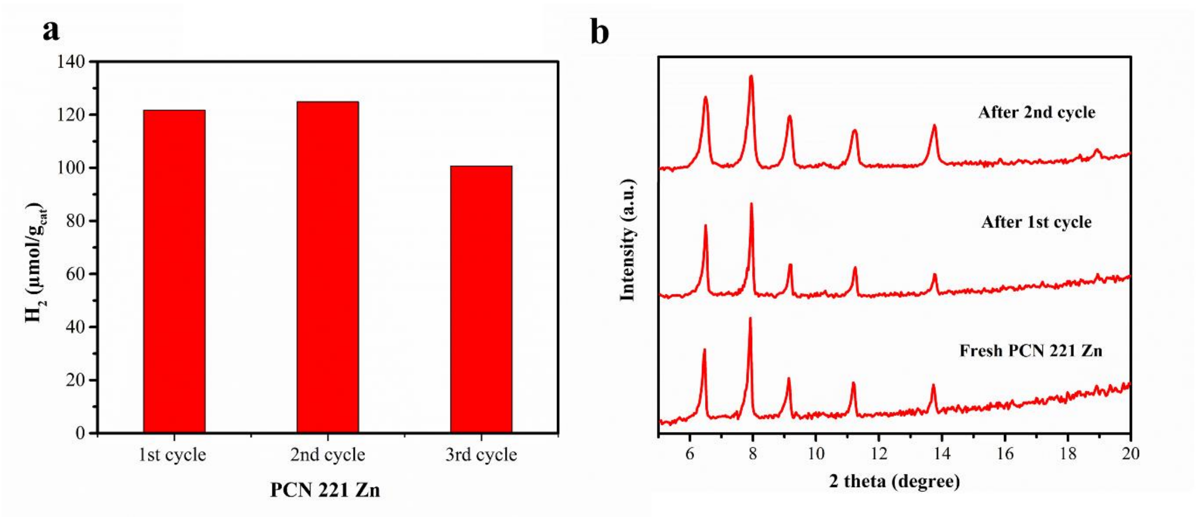

Figure S20. (a) 1st , 2nd and 3rd cycle of the PCN 221 Zn MOF for the photocatalytic production of H_2 (μmol/g_{cat} *vs*. time) monitored for 72 h under the following reaction conditions: 1 mg of MOF, deaerated H_2O: CH_3CN: TEOA (1:8:2 v/v); λ: 405 nm LED source. For the 2nd and 3rd cycle the solid was separated from the solution by centrifugation at 7000 rpm, washed with CH_3CN (twice), and re-irradiated (2nd cycle and 3rd cycle) with the deaerated H_2O: CH_3CN: TEOA (1:8:2 v/v). (b) PXRD pattern of the PCN 221 Zn sample dried after each cycle, obtained from separate experiments.

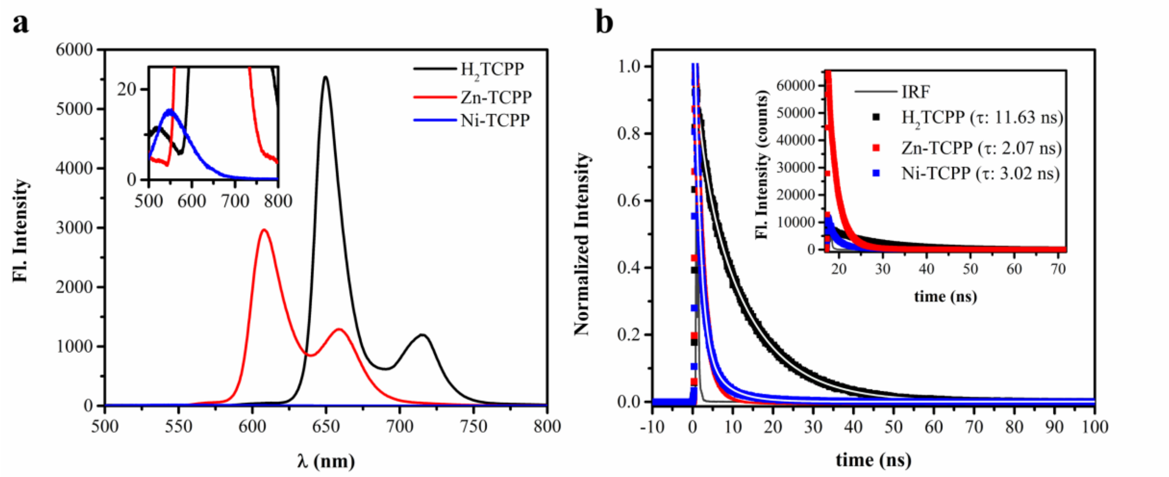

Figure S21. (a) Emission spectra of the ~2.65 µM of M-TCPP linkers in deaerated DMF solution and excited at 427 nm, (b) The normalized fluorescence decay curves (inset showing actual Fl. signals vs time trace) measured for the ~2.65 µM of M-TCPP linkers under deaerated DMF excited at the wavelength of 372 nm (a 495 nm long pass filter is used to collect the trace). The decay curves were fitted to a mono-exponential ($y = y_0 + A_1 e^{\left(\frac{-(x-x_0)}{\tau_1}\right)}$) function for H_2TCPP, and Zn-TCPP and a biexponential decay $y = y_0 + A_1 e^{\left(\frac{-(x-x_0)}{\tau_1}\right)} + A_2 e^{\left(\frac{-(x-x_0)}{\tau_2}\right)}$ function for Ni-TCPP. $y_0 = 0$, and τ_{av} for Ni-TCPP is the average lifetime, is calculated according to the equation $\tau_{av} = \sum_{i=1}^{2} \frac{A_i \tau_i^2}{A_i \tau_i}$.

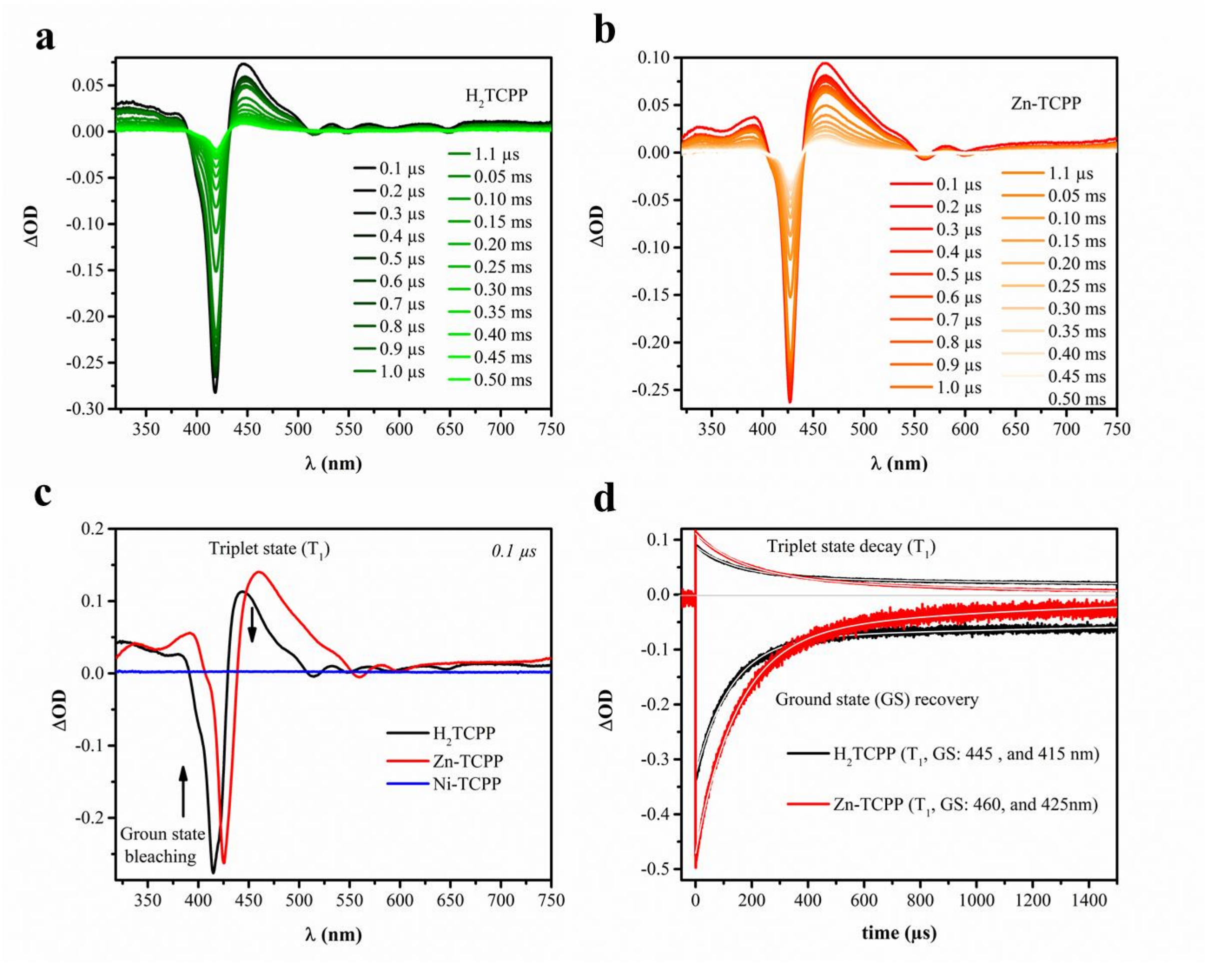

Figure S22. ns-transient absorption spectra of 13 µM M-TCPP linkers: (a) H_2TCPP and (b) Zn-TCPP in deaerated DMF at a time delay of 0.1-500 µs and (c) a comparison of ΔOD of 13 µM Zn-TCPP linkers with 13µM of H_2 TCPP and Ni-TCPP linkers at a time delay of 100 ns, (d) kinetic traces of the triplet state at 460 nm (Zn-TCPP) and 445 nm (H_2TCPP), and respective ground state recoveries, where $\lambda_{ex\ (pump)}$ = 410 nm. Kinetic traces are fitted with biexponential decay functions ($y = y_0 + A_1 e^{(\frac{-x}{\tau_1})} + A_2 e^{(\frac{-x}{\tau_2})}$), where $y_0 = 0$. The red, black, and blue line denotes Zn-TCPP, H_2TCPP, and Ni-TCPP, respectively.

Table S4. Excited state (triplet) photophysical data of Zn-TCPP and H_2TCPP in deaerated DMF solution.

**Linker**	**triplet state (T_1^*) λ (nm)**	**Triplet state decay**					**Ground state Recovery**				
		**A_i**	**B_i^b %**	**τ_i (µs)**	**K_i (µs^{-1})**	**R^2**	**A_i**	**B_i^b %**	**τ_i (µs)**	**K_i (µs^{-1})**	**R^2**
H_2TCPP[a]	445	0.06	8	149	6.7×10^{-3}	0.99	0.25	7	124	8.0×10^{-3}	0.99
		0.03	92	3556	2.8×10^{-4}		0.08	93	5473	1.8×10^{-4}	
Zn-TCPP[a]	460	0.08	29	167	6.0×10^{-3}	0.99	0.39	39	145	6.9×10^{-3}	0.99
		0.03	71	911	1.1×10^{-3}		0.07	61	1246	8.0×10^{-4}	
Ni-TCPP[c]	-	-	-	-	-	-	-	-	-	-	-

[a] bi-exponential decay functions ($y=y_0+A_1 e^{(\frac{-x}{\tau_1})}+A_2 e^{(\frac{-x}{\tau_2})}$) is used to fit the trace, where y_0= 0. [b] B_i is the integral contribution of the i th component as calculated according to equation ($B_i = \frac{A_i\tau_i}{\sum_{i=1}^{2} A_i\tau_i}$). No transient absorption signal was observed within the time resolution.

Stern Volmer experiments

For the Stern-Volmer titrations, ~2.65 µM Zn-TCPP or 2.75 µM Zn-TCPPOMe solutions in deaerated DMF were prepared. A variable amount of quencher (Zr_{12} or TEOA) under an argon atmosphere is added to the linker solution under a glove box.

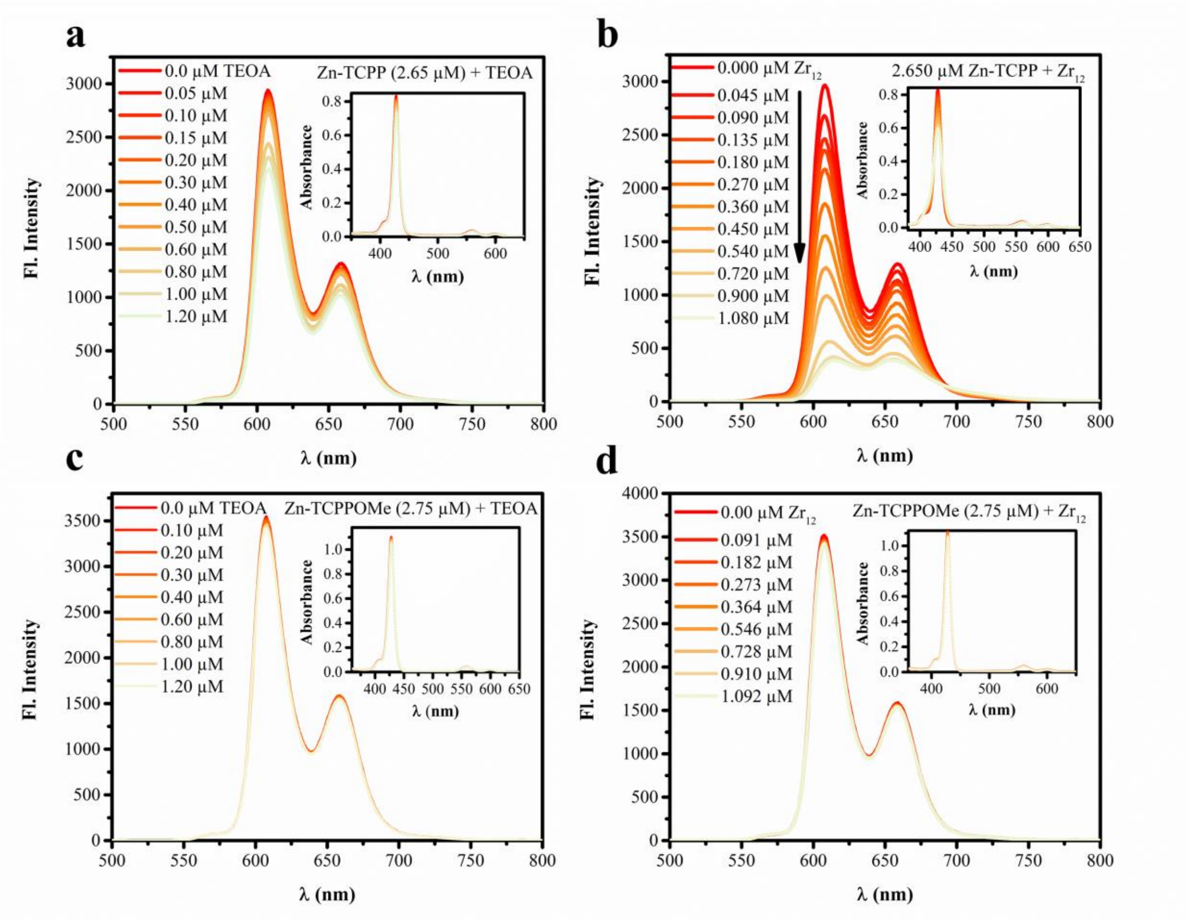

Figure S23. Steady-state emission spectra at λ_{ex}: 427 nm (inset shows respective steady state UV-visible abs. spectra) of Ar purged DMF solution of ~2.65 µM of (a-b) Zn-TCPP and (c-d) 2.75 µM of Zn-TCPPOMe in the presence of (a, c) sacrificial donor: TEOA, [0 µM] → [1.20 µM], and (b, d) acceptor: Zr_{12}, [0 µM] → [1.080 µM].

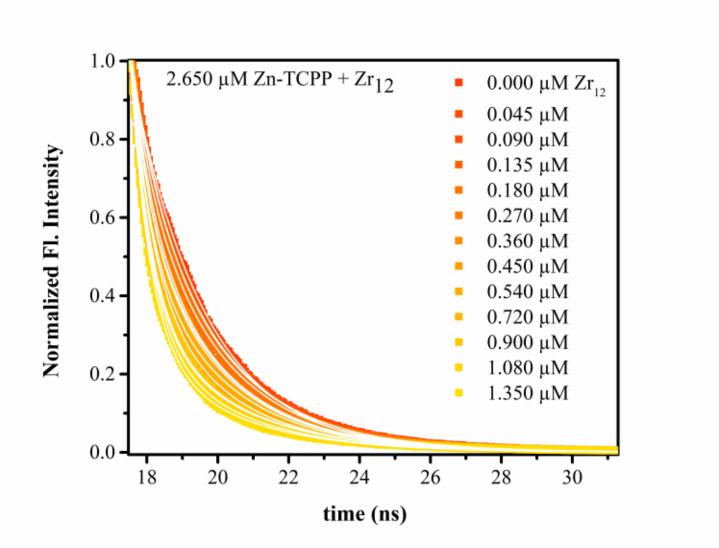

Figure S24. The normalized fluorescence decay curves were measured for the ~2.65 µM of Zn-TCPP linkers under deaerated DMF excited at the wavelength of 372 nm at various concentrations of Zr_{12} cluster ([0 µM] → [1.350 µM]). The decay curves were acquired by using a 495 nm long pass filter and fitted to a mono-exponential function ($y{=}y_0{+}A_1 e^{\left(\frac{-(x-x_0)}{\tau_1}\right)}$) at 0 µM of Zr_{12} and a biexponential decay function ($y{=}y_0{+}A_1 e^{\left(\frac{-(x-x_0)}{\tau_1}\right)}{+}A_2 e^{\left(\frac{-(x-x_0)}{\tau_2}\right)}$) at [0.045 µM] to [1.350 µM]) of Zr_{12}.

Table S5. The multiexponential fitting parameters (monoexponential and biexponential) derived from time-resolved emission spectroscopy (singlet state decay).

[Zr_{12}] (µM)	A_i		x_0	τ_i *(ns)*		Adjacnt R^2	τ_{av} [b](ns)
	A_1	A_2	f_i	τ_1	τ_2		
0[a]	0.978	-	17.553	2.072±0.002	-	0.99899	2.072
0.045	0.786	0.219	17.553	2.328±0.006	0.770±0.013	0.99963	2.196
0.090	0.718	0.295	17.553	2.407±0.009	0.926±0,014	0.99961	2.205
0.135	0.630	0.379	17.553	2.335±0.006	0.643±0.005	0.99951	2.094
0.180	0.740	0.273	17.553	2.303±0.005	0.631±0.007	0.99959	2.149
0.270	0.651	0.359	17.553	2.271±0.005	0.517±0.004	0.99946	2.075
0.360	0.614	0.394	17.553	2.303±0.006	0.565±0.004	0.99943	2.067
0.450	0.477	0.559	17.501	2.355±0.009	0.608±0.003	0.99926	1.949
0.540	0.554	0.448	17.553	2.327±0.006	0.516±0.003	0.99930	2.051
0.720	0.375	0.654	17.501	2.561±0,011	0.560±0,002	0.99898	2.009
0.900	0.376	0.656	17.501	2.587±0.012	0.589±0.003	0.99892	2.019
1.080	0.333	0.703	17.501	2.512±0.010	0.566±0.002	0.99901	1.885
1.350	0.295	0.741	17.501	2.558±0.013	0.552±0.002	0.99898	1.853

The photoluminescence decay curves at 0 µM of Zr_{12}, and at [0.045 µM] to [1.350 µM]) of Zr_{12} were fitted to a [a]mono-exponential ($y{=}y_0{+}A_1 e^{\left(\frac{-(x-x_0)}{\tau_1}\right)}$), and a biexponential decay functions ($y{=}y_0{+}A_1 e^{\left\{\frac{-(x-x_0)}{\tau_1}\right\}}{+}A_2 e^{\left(\frac{-(x-x_0)}{\tau_2}\right)}$), respectively. $y_0 = 0$, and [b] τ_{av} is the average lifetime, is calculated according to equation $\tau_{av} = \sum_{i=1}^{2} \frac{A_i \tau_i^2}{A_i \tau_i}$.

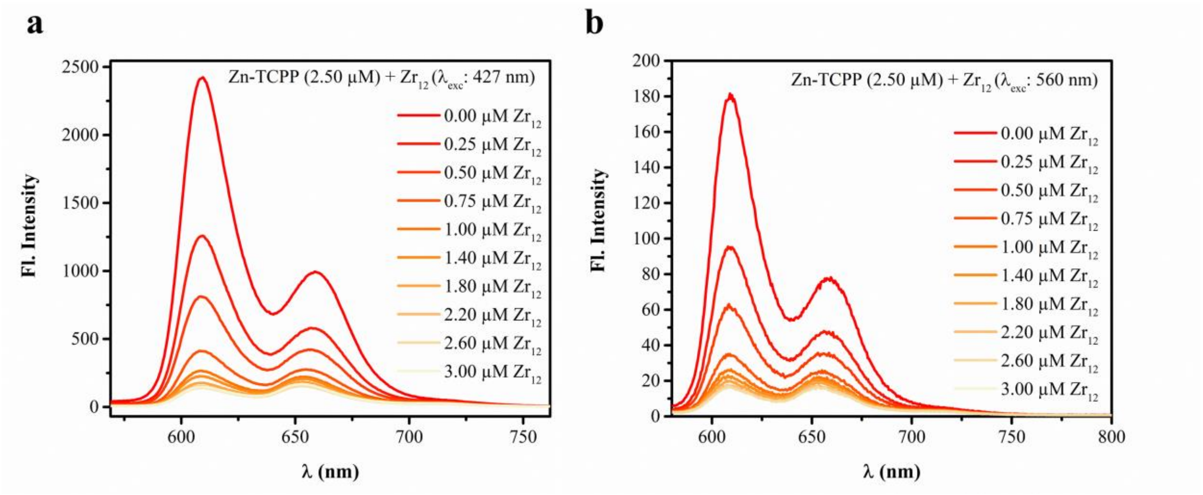

Figure S25. Steady-state emission spectra of Ar purged DMF solution of ~2.65 µM of Zn-TCPP in the presence of Zr_{12} ([0.0 µM] to [3.0 µM]) at λ_{ex} of (a) 427 nm, and (b) 560 nm.

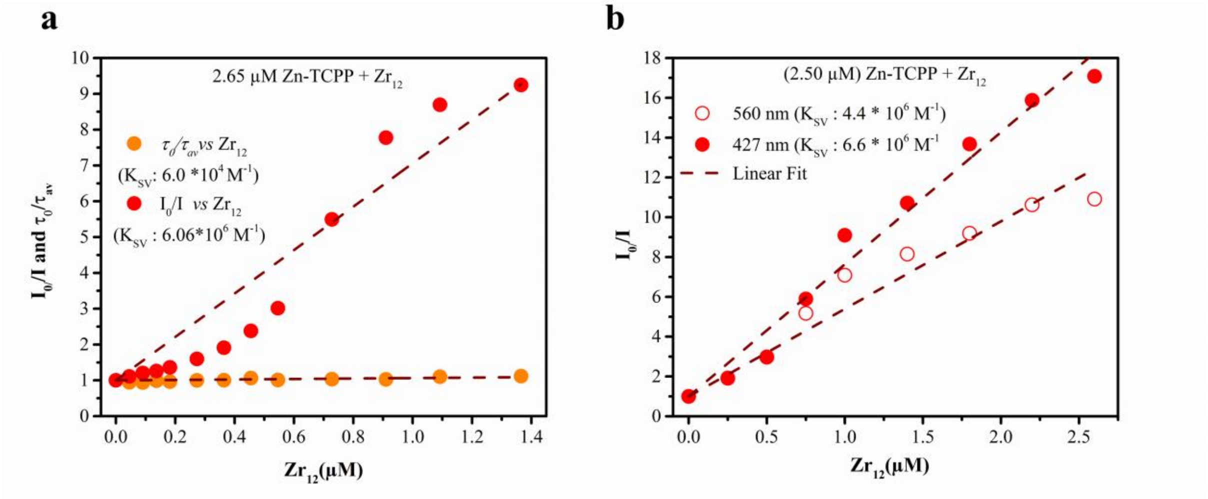

Figure S26. (a) Plots of I_0/I (red) and τ_0/τ (orange) for the Ar purged DMF solution of 2.65 µM of Zn-TCPP as a function of the concentration of Zr_{12} cluster ([0.000 µM] to [1.350 µM]) obtained from the emission quenching experiments (λ_{ex} of 427 nm for I_0/I, and λ_{ex} of 372 nm for τ_0/τ with a 495 nm long pass filter). (b) The plots of I_0/I for the same Zn-TCPP solution as a function of the concentration of Zr_{12} cluster (([0.0 µM] to [3.0 µM]) obtained from the emission quenching experiments at λ_{ex} of 427 nm (solid circle), and 560 nm (hollow circle). A linear fitting function ($y = 1 + bx$) is used.

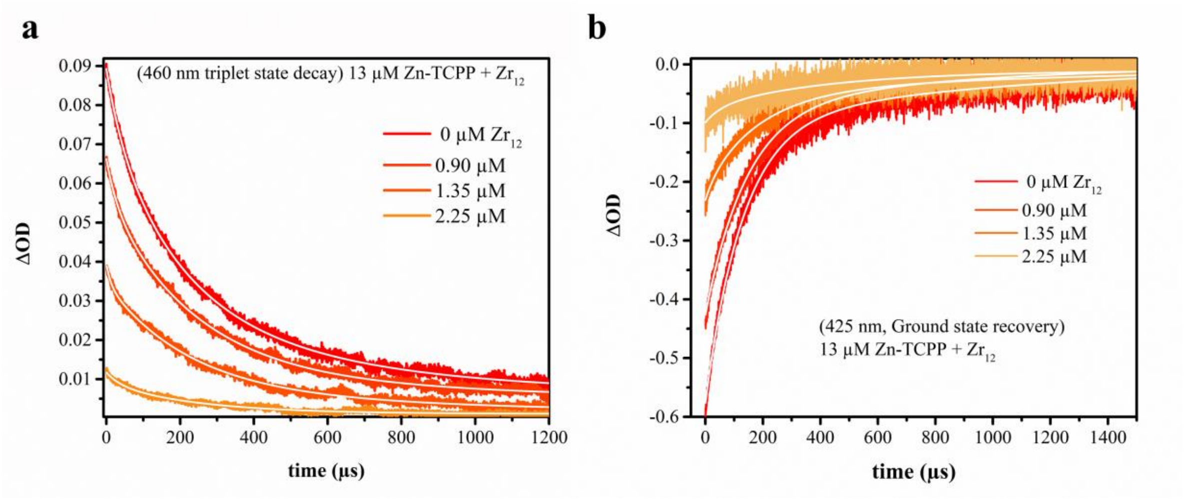

Figure S27. The kinetic profiles of the (a) triplet state decay at 460 nm and (b) ground state recovery observed at 460 and 425 nm, respectively, for 13 μM Zn-TCPP linkers in deaerated DMF solution, were acquired from ns-transient absorption spectroscopy as a function of Zr_{12} cluster concentration ([0 μM] → [2.25 μM]), with λ_{ex}(pump) = 410 nm. [a] a biexponential function ($y = y_0 + A_1 e^{(\frac{-x}{\tau_1})} + A_2 e^{(\frac{-x}{\tau_2})}$) at Zr_{12} concentration (0, 0.90, and 2.25 μM), and [b] a triexponential function ($y = y_0 + A_1 e^{(\frac{-x}{\tau_1})} + A_2 e^{(\frac{-x}{\tau_2})} + A_3 e^{(\frac{-x}{\tau_3})}$) at Zr_{12} concentration (1.35 μM) was used to fit the decay and recovery curve. where $y_0 = 0$.

Table S6. Excited state (triplet) photophysical data of Zn-TCPP in Ar-purged DMF at various concentrations of Zr_{12} cluster.

**[Zr_{12}]/ μM**	**Triplet state decay at 460 nm**					**Ground state recovery at 425 nm**				
	**A_i**	**B_i^c (%)**	**τ_i (μs)**	**K_i (μs^{-1})**	**R^2**	**A_i**	**B_i^c (%)**	**τ_i (μs)**	**K_i (μs^{-1})**	**R^2**
0[a]	0.077	29	167	6.0×10^{-3}	0.999	0.39	39	145	6.87×10^{-3}	0.995
	0.035	71	911	1.1×10^{-3}		0.07	61	1246	8.02×10^{-4}	
0.90[a]	0.048	29	193	5.0×10^{-3}	0.997	0.17	38	117	8.0×10^{-3}	0.983
	0.014	71	1597	0.6×10^{-3}		0.05	62	674	1.0×10^{-3}	
1.35[b]	0.005	1	12	8.5×10^{-2}	0.997	0.03	3	45	22×10^{-3}	0.983
	0.029	41	229	4.0×10^{-3}		0.10	38	199	5.0×10^{-3}	
	0.006	58	1536	1.0×10^{-3}		0.02	59	1437	1.0×10^{-3}	
2.25[a]	0.003	5	38	2.6×10^{-2}	0.952	0.03	6	56	1.7×10^{-2}	0.995
	0.008	95	246	4.0×10^{-3}		0.06	94	429	2.3×10^{-3}	

[a] a biexponential function ($y = y_0 + A_1 e^{(\frac{-x}{\tau_1})} + A_2 e^{(\frac{-x}{\tau_2})}$), and [b] a triexponential function ($y = y_0 + A_1 e^{(\frac{-x}{\tau_1})} + A_2 e^{(\frac{-x}{\tau_2})} + A_3 e^{(\frac{-x}{\tau_3})}$) was used to fit the decay and recovery curve. where $y_0 = 0$, and [c] B_i is the integral contribution of the i th component as calculated according to equation $B_i = \frac{A_i \tau_i}{\sum_{i=1}^{3} A_i \tau_i}$.

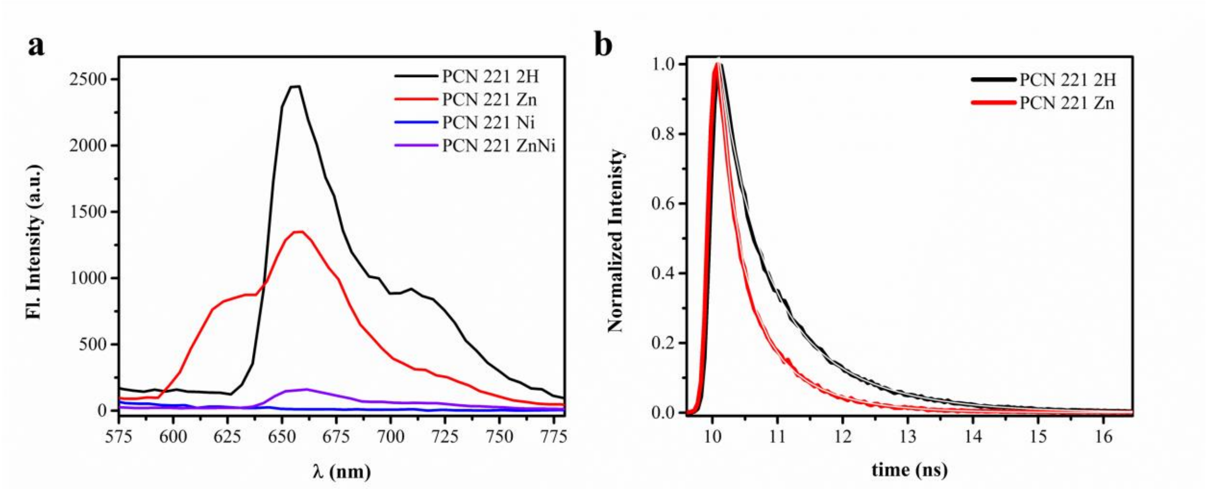

Figure S28. (a) Steady-state emission spectra of the PCN 221 M MOFs (66-78 µg) dispersed in deaerated DMF solutions. (b) Decay curves of the single-state emission for PCN 221 M MOFs (~350 µg) in deaerated DMF. Excitation wavelength: 372 nm, with a 495 nm long-pass filter. The curves were fitted to a mono-exponential function ($y = y_0 + A_1 e^{(\frac{-(x-x_0)}{\tau_1})}$).

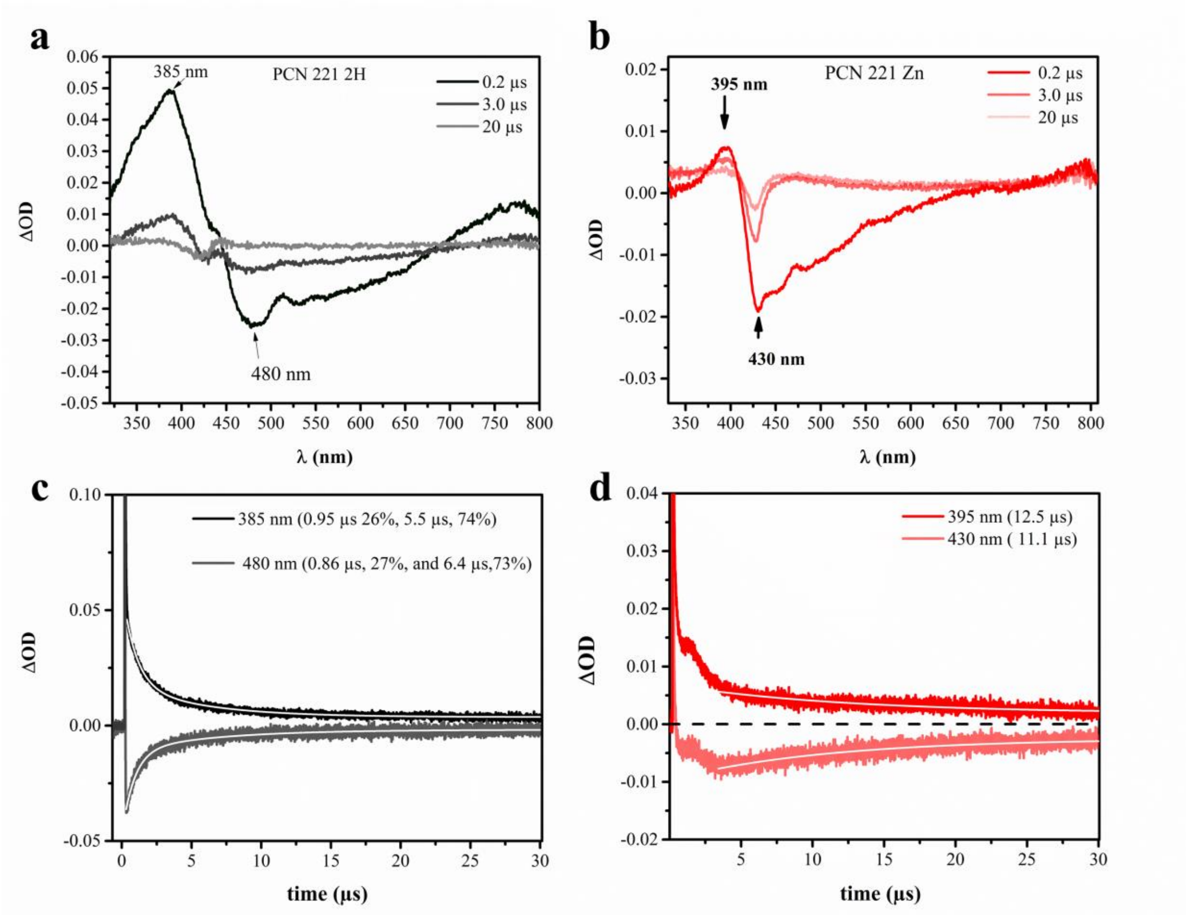

Figure S29. Transient absorption spectra of the (a) PCN 221 2H and (b) PCN 221 Zn MOFs (0.1 mg/4mL) dispersed in deaerated DMF at various time delays (0.2, 3.0, and 20 µs), and kinetic traces for (c) PCN 221 2H at 385 nm, and 480 nm, and (d) PCN 221 Zn at 395 nm, and 430 nm when $\lambda_{ex(pump)}$ =410 nm. A biexponential decay function ($y = y_0 + A_1 e^{\{\frac{-(x-x_0)}{\tau_1}\}} + A_2 e^{\left(\frac{-(x-x_0)}{\tau_2}\right)}$, $y_0 = 0$) is used to fit the kinetic curve of PCN 221 2H. while

to estimate the long-lived 2nd component only in PCN 221 Zn, a mono-exponential function ($y = y_0 + A_1 e^{(\frac{-(x-x_0)}{\tau_1})}$, $y_0 = 0$) is used.

MOF sample preparation for the transient absorption measurements with better quality:

Utilizing a recently reported method to enhance data quality, as detailed in.[8] Approximately 3 mg of PCN 221 M MOF (M = 2H, Zn) and 100 mg of NH_2-terminated polyethylene glycol (PEG-NH_2, molecular weight 2000) were combined in a 4 mL vial for sample preparation. Subsequently, 3 mL of anhydrous DMF was introduced, and the mixture was sonicated for 5 minutes. The resultant suspension was subsequently centrifuged at 7000 rpm for 30 seconds. A 0.5 mL aliquot of the resulting supernatant was transferred to a quartz optical cell and diluted with 4 mL of DMF to achieve an absorbance level (Soret band) of 2.0 or lower. The prepared solutions were sealed with a rubber septum, purged with argon gas for 30 minutes, and then used in the experiments. Additionally, 100 µL of TEOA is added to perform the transient absorption measurement of PCN 221 Zn in the presence of a donor.

Table S7. Excited state photophysical data for PCN 221 Zn and PCN 221 2H were obtained through transient absorption spectroscopy in Ar-purged DMF under various conditions.

**Linker**	**Excited state**	**Excited state decay**				**Ground state Recovery**			
	**λ (nm)**	**A_i**	**B_i (%)**	**τ_i (µs)**	**K_i (µs^{-1})**	**A_i**	**B_i (%)**	**τ_i (µs)**	**K_i (µs^{-1})**
PCN 221 2H[a]	385	0.028	26	0.95	1.05	0.025	27	0.86	1.16
		0.014	74	5.50	0.18	0.009	73	6.40	0.16
PCN 221 2H[b]	390	0.005	19	0.22	4.54	0.022	27	0.36	2.77
		0.002	81	2.03	0.49	0.007	73	3.14	0.32
PCN 221 Zn[a, c]	395	-	-	-	-	-	-	-	-
		0.004	100	12.5	0.08	0.005	100	11.0	0.09
PCN 221 Zn[b]	400	0.007	14	0.37	2.70	0.019	13	0.34	2.94
		0.003	86	5.54	0.18	0.010	87	4.62	0.21
PCN 221 Zn[b]	400	0.002	14	0.21	4.76	0.018	15	0.19	5.26
+ 150 mM TEOA		0.001	86	2.18	0.45	0.067	85	3.00	0.33

[a] Measurements for the MOF samples were carried out [a] without any modification and [b] with PEG-NH_2 modified. A biexponential decay function ($y = y_0 + A_1 e^{\{\frac{-(x-x_0)}{\tau_1}\}} + A_2 e^{(\frac{-(x-x_0)}{\tau_2})}$, $y_0 = 0$) is used for all the samples. [c]Exceptionally, a mono-exponential function ($y = y_0 + A_1 e^{(\frac{-(x-x_0)}{\tau_1})}$, $y_0 = 0$) is used to estimate the long-lived 2nd component only in unmodified PCN 221 Zn. [d] τ_{av} is the average lifetime, is calculated according to equation $\tau_{av} = \sum_{i=1}^{2} \frac{A_i \tau_i^2}{A_i \tau_i}$.

Cyclic voltammetry

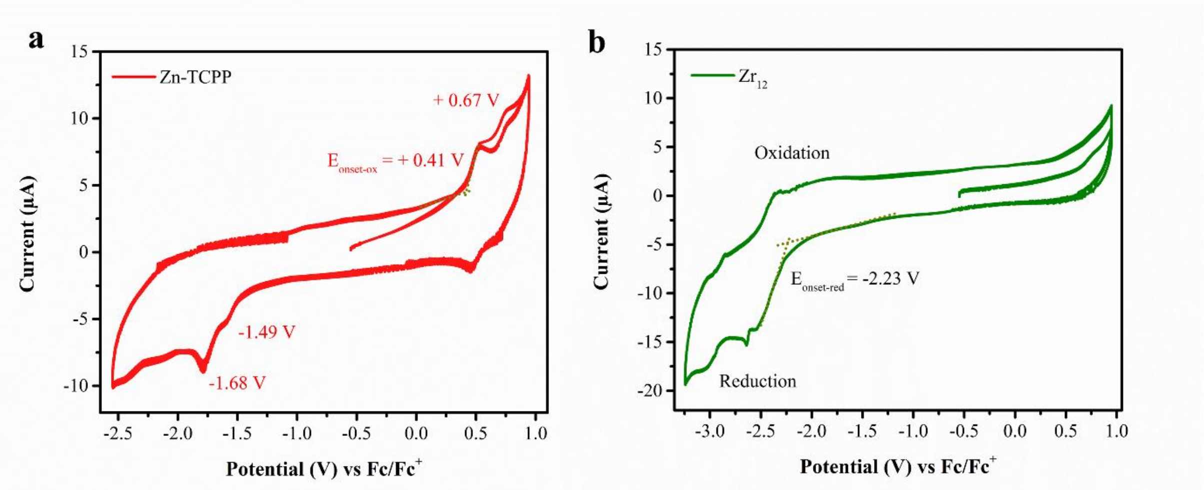

Figure S30. Cyclic voltammograms of (a) Zn-TCPP at 100 mVs^{-1} and (b) Zr_{12} at 100 mVs^{-1} (Experimental conditions: DMF, 0.1 M nBu_4NPF_6 supporting electrolyte, 0.25 mM analyte, glassy carbon-working electrode, Pt-wire counter electrode, Ag/AgCl quasi-reference electrode) referenced against Fc/Fc$^+$.

Energy level of HOMO and LUMO in Zn-TCPP and reduction potential of the Zr_{12} cluster (*vs* NHE)

According to the results of cyclic voltammetry, and the steady state absorption spectroscopy the HOMO, LUMO (1, Q-band) and the LUMO (2, Soret-band) orbits are approximated by the following formulas:[9,10]

$$E_{Fc/Fc+}\ vs.\ NHE = E_{Fc/Fc+}\ vs.\ SCE + 0.241\ V = 0.45\ V + 0.24\ V = 0.69\ V$$

Therefore,

E_{HOMO} *vs* NHE = ($E_{onset\text{-}ox}$ *vs* Fc/Fc$^+$ + 0.69) = (0.41 *vs* Fc/Fc$^+$ + 0.69) = +1.10 V *vs* NHE

$E_{Q\text{-band}}$ (optical) =1240/$\lambda_{Q\ band\ onset}$ = 1240/621 eV = 1.99 eV

$E_{LUMO\ (1)}$ *vs* NHE = (E_{HOMO} *vs* NHE- $E_{Q\text{-band}}$ (optical)) = (+1.10 -1.99) = - 0.89 V *vs* NHE

$E_{Soret\text{-}band}$ (optical) = 1240/$\lambda_{Soret\text{-}band\ onset}$ = 1240/438 = 2.83 eV

$E_{LUMO\ (2)}$ *vs* NHE = (E_{HOMO} *vs* NHE -$E_{Soret\text{-}band\ onset}$ (optical)) = (+ 1.10 - 2.83) = - 1.73 V *vs* NHE

Whereas, estimated potential for reduction of the Zr_{12} cluster ($E_{onset\text{-}red}$ *vs* Fc/Fc$^+$ + 0.69) = (-2.23 *vs* Fc/Fc$^+$ + 0.69) = -1.54 V *vs* NHE, which is very close to the CB potential of ZrO_2 (-1.78 V vs NHE)[11]

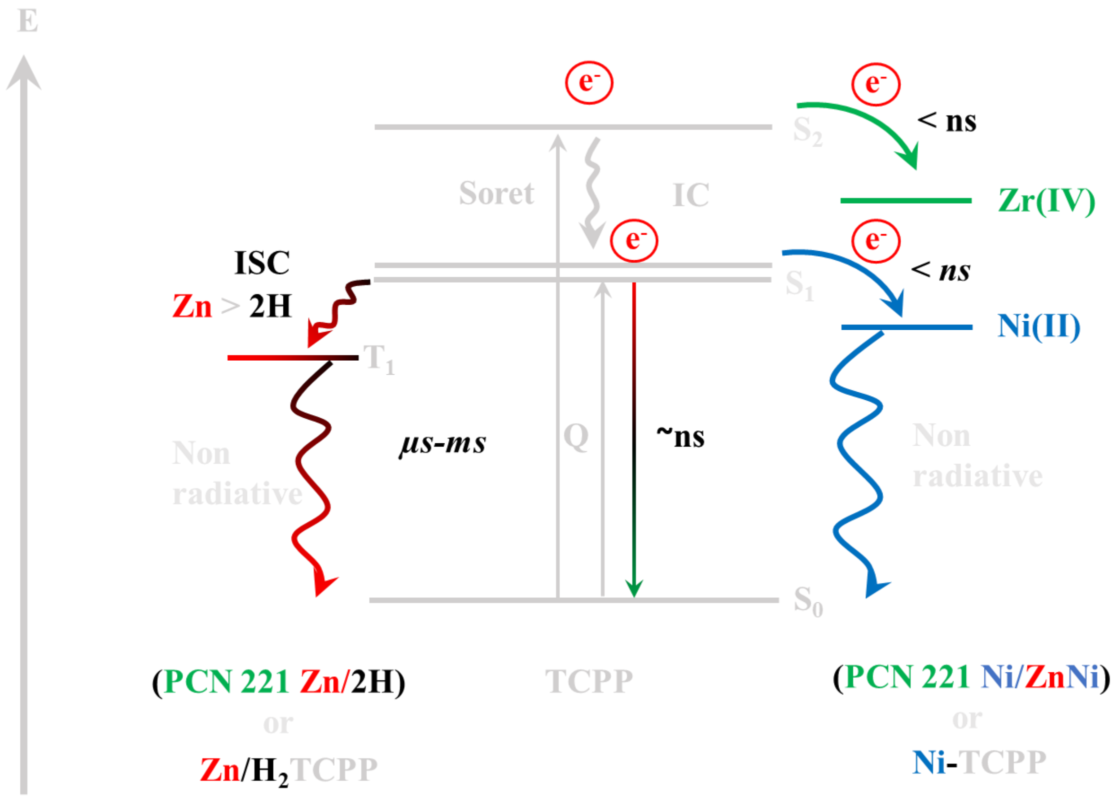

Figure S31. Schematic energy level diagram showing the M-TCPP linker's working principle when it is free and coordinated to the Zr_8 node (green) in the MOF after exposure to various wavelengths (Soret and Q band) and variations of the porphyrin's central metal atom (2H: black, Zn: red, and Ni: blue). The energy level position is considered according to the literature reported earlier. [12–14]

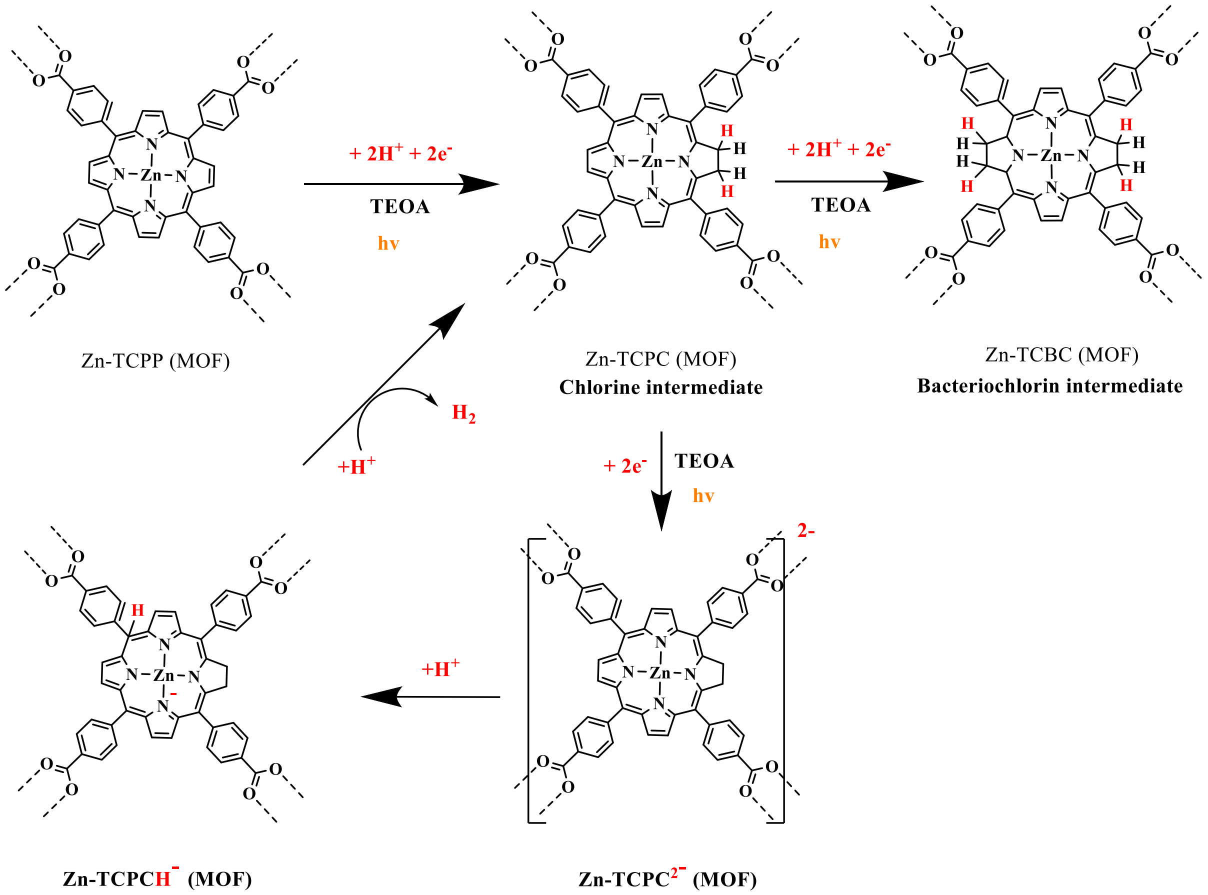

Figure 32. Photoreduction of Zn-TCPP in the MOF as observed in aq. homogenous condition reported earlier[15] and a HER under visible-light based on the formed zinc chlorin sensitizer (Zn-TCPC) reversibly forming a chlorin–phlorin anion intermediate (Zn-TCPCH$^-$) acting as a hydride supplying catalyst.[16]

References

[1] D. Feng, Z. Gu, J. Li, H. Jiang, Z. Wei, H. Zhou, *Angewandte Chemie International Edition* **2012**, *51*, 10307.

[2] D. Feng, W. C. Chung, Z. Wei, Z. Y. Gu, H. L. Jiang, Y. P. Chen, D. J. Darensbourg, H. C. Zhou, *J Am Chem Soc* **2013**, *135*, 17105.

[3] J. Ji, F. Liu, W. Yang, M. Tan, W. Luo, S. Yin, *ChemCatChem* **2020**, *12*, 4331.

[4] M. Mojiri-Foroushani, H. Dehghani, N. Salehi-Vanani, *Electrochim Acta* **2013**, *92*, 315.

[5] D. Feng, H. L. Jiang, Y. P. Chen, Z. Y. Gu, Z. Wei, H. C. Zhou, *Inorg Chem* **2013**, *52*, 12661.

[6] Y. Zhang, F. de Azambuja, T. N. Parac-Vogt, *Catal Sci Technol* **2022**, *12*, 3190.

[7] G. Kickelbick, P. Wiede, U. Schubert, *Inorganica Chim Acta* **1999**, *284*, 1.

[8] Y. Benseghir, A. Solé-Daura, D. R. Cairnie, A. L. Robinson, M. Duguet, P. Mialane, P. Gairola, M. Gomez-Mingot, M. Fontecave, D. Iovan, B. Bonnett, A. J. Morris, A. Dolbecq, C. Mellot-Draznieks, *J Mater Chem A Mater* **2022**, *10*, 18103.

[9] D. A. Jose, A. D. Shukla, G. Ramakrishna, D. K. Palit, H. N. Ghosh, A. Das, *J Phys Chem B* **2007**, *111*, 9078.
[10] H.-C. Chen, J. N. H. Reek, R. M. Williams, A. M. Brouwer, *Physical Chemistry Chemical Physics* **2016**, *18*, 15191.
[11] K. Kalyanasundaram, *Coord Chem Rev* **1998**, *177*, 347.
[12] P. M. Stanley, K. Hemmer, M. Hegelmann, A. Schulz, M. Park, M. Elsner, M. Cokoja, J. Warnan, *Chem Sci* **2022**, *13*, 12164.
[13] A. Rosa, G. Ricciardi, E. J. Baerends, M. Zimin, M. A. J. Rodgers, S. Matsumoto, N. Ono, *Inorg Chem* **2005**, *44*, 6609.
[14] T. Kobayashi, K. D. Straub, P. M. Rentzepis, *Photochem Photobiol* **1979**, *29*, 925.
[15] C. D. Windle, M. W. George, R. N. Perutz, P. A. Summers, X. Z. Sun, A. C. Whitwood, *Chem Sci* **2015**, *6*, 6847.
[16] S. Salzl, M. Ertl, G. Knör, *Physical Chemistry Chemical Physics* **2017**, *19*, 8141.
